# Drug Repurposing Investigation for Combating Ebola Virus Disease: Database Mining, Docking Calculations, Molecular Dynamics, and Density Functional Theory Study

**DOI:** 10.1002/open.202500348

**Published:** 2025-09-02

**Authors:** Alaa H. M. Abdelrahman, Gamal A. H. Mekhemer, Peter A. Sidhom, Mohamed A. El‐Tayeb, Shahzeb Khan, Mahmoud A. A. Ibrahim

**Affiliations:** ^1^ Computational Chemistry Laboratory Chemistry Department Faculty of Science Minia University Minia 61519 Egypt; ^2^ Department of Pharmaceutical Chemistry Faculty of Pharmacy Tanta University Tanta 31527 Egypt; ^3^ Department of Botany and Microbiology College of Science King Saud University P.O. Box 2455 Riyadh 11451 Saudi Arabia; ^4^ Centre for Pharmaceutical Engineering Science Faculty of Life Science School of Pharmacy and Medical Sciences University of Bradford Bradford BD7 1DP UK; ^5^ Department of Engineering College of Engineering and Technology University of Technology and Applied Sciences Nizwa 611 Sultanate of Oman; ^6^ School of Health Sciences University of KwaZulu‐Natal, Westville Campus Durban 4000 South Africa

**Keywords:** density functional theory computations, docking calculations, drug repurposing, Ebola virus VP35, molecular dynamics simulations

## Abstract

Ebola virus (EBOV), one of the deadliest diseases, is responsible for infecting individuals with hemorrhagic fever syndrome, which remains an ongoing worldwide health concern. The extremely deadly nature and virulence of EBOV illness illuminate the imperative need to evolve effective curative agents. Viral protien (VP35) acts as an Achilles heel for EBOV reproduction and also interacts with numerous human proteins, which leads to impairing the immune system. Herein, the DrugBank database, containing >14000 investigational and approved drugs, is mined to hunt prospective inhibitors toward VP35 utilizing various computational approaches. Docking technique performance is initially validated to predict the VP35‐inhibitor binding pose upon the accessible experimental data. Molecular dynamics simulations (MDS) are then conducted in triplicate on the top potent drug candidates, followed by binding energy (Δ*G*_binding_) estimations using molecular mechanics/generalized Born surface area (MM/GBSA) approach. Upon MM/GBSA//250 ns MDS, DB14875 and DB07800 revealed better binding energy against VP35 than 1D9, reference inhibitor, with Δ*G*_binding_ values of −36.6, −35.6, and −29.3 kcal mol^−1^, respectively. Post‐MD analyses demonstrate great stability for the identified drug candidates complexed with VP35 over 250 ns MDS. Ultimately, the density functional theory computations are executed, and their outcomes elucidate favorable molecular reactivity of the identified drug candidates. Conclusively, these findings suggest promising inhibitors for VP35, warranting further experimental assays.

## Graphical abstract

DrugBank database is mined to hunt prospective Ebola virus (EBOV) VP35 inhibitors utilizing docking calculations, molecular dynamics, molecular mechanics/generalized Born surface area binding energy calculations, and density functional theory computations. The results suggest that DB14875 and DB07800 can serve as effective inhibitors of VP35, indicating their potency to be developed into anti‐EBOV medications.

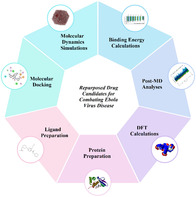

## 1 Introduction

Ebola virus (EBOV) is an enveloped RNA virus that resembles a thread and is a member of the *Filoviridae* family. EBOV is a lethal pathogen liable for hemorrhagic fever and has mainly garnered notoriety in the past ten years.^[^ ^1^ ^]^ EBOV causes respiratory and gastrointestinal problems, various organ failure, and hypovolemic shock.^[^ ^2^ ^,^ ^3^ ^]^ The usual death rate of EBOV ranges from 50% to 90%, which presents a severe risk to public health.^[^ ^4^ ^]^ The EBOV pandemic was the most widespread in West Africa from 2013 to 2016, with 28 652 infections, resulting in 11 325 deaths.^[^ ^5^ ^]^ The necessity for discovering efficient medication to stop the spread of EBOV has been highlighted due to the recent outbreaks in Guinea in 2021.^[^ ^6^ ^]^

Seven structural proteins (SPs) are encoded by the EBOV genome, which is 19 kb in size. These SPs include RNA‐dependent RNA polymerase (L), nonstructural secretory glycoprotein (sGP), small secretory glycoprotein (ssGP), viral proteins (VP35, VP30, and VP24), nucleoproteins (NPs), matrix proteins (VP40), and glycoproteins (GPs). ^[^ 7, 8, 9 ^]^ The main targets of EBOV reproduction are dendritic cells (DCs), macrophages, and antigen‐presenting cells (APCs) that are presented at the contagion location.^[^ ^10^ ^]^ Among these targets, VP35 is a multifunctional target that plays an essential role in the pathogenesis of EBOV and its life cycle; thus, VP35 is a charming druggable target.^[^ ^11^ ^]^ VP35, a cofactor for RNA polymerase, is essential for both viral assembly and RNA synthesis. Moreover, VP35 plays a crucial role in the ability of EBOV to evade the host immune response by inhibiting the production of type I interferons (IFN‐α/β), which are central to initiating antiviral defenses.^[^ ^12^ ^]^ Under normal circumstances, viral RNA is recognized by host pattern recognition receptors, such as RIG‐I and MDA5, leading to the activation of downstream signaling pathways and induction of IFN‐α/β. ^[^ ^13^ ^]^ These interferons then stimulate the expression of numerous interferon‐stimulated genes that restrict viral replication and spread. Consequently, targeting VP35 is a promising strategy to deactivate the various stages of the EBOV life cycle, hence limiting the spread of infection. It is worth noting that VP35 is a versatile protein that remains well‐conserved across various filoviruses, including other *Filoviridae* family members, such as Sudan virus (SUDV), Marburg virus (MARV), and Bundibugyo virus (BDBV).^[^ ^14^ ^]^ VP35 is essential in evading host immune defenses and supporting viral replication is shared among these viruses. As such, discovering small‐molecule inhibitors that target EBOV VP35 could contribute to the development of broad‐spectrum antivirals effective against multiple filoviruses. Recently, a couple of EBOV therapies were endorsed by the Food and Drug Administration (FDA).^[^ ^15^ ^]^ Ansuvimab (ebangaTM) prevents viral entrance and halts replication. Inmazeb (REGN‐EB3) contains three monoclonal antibodies—namely maftivimab, altovimab, and odesivimab‐ebgn—that prevent the attachment and penetration of viruses into cells by inhibiting viral GP.^[^ 16, 17, 18 ^]^ However, these drugs are confined to pain alleviation and palliative care to address dehydration and insufficient oxygen supply.^[^ ^19^ ^]^ This highlights the urgent need to discover efficacious antiviral therapies that combat this deadly illness.

**Figure 1:**
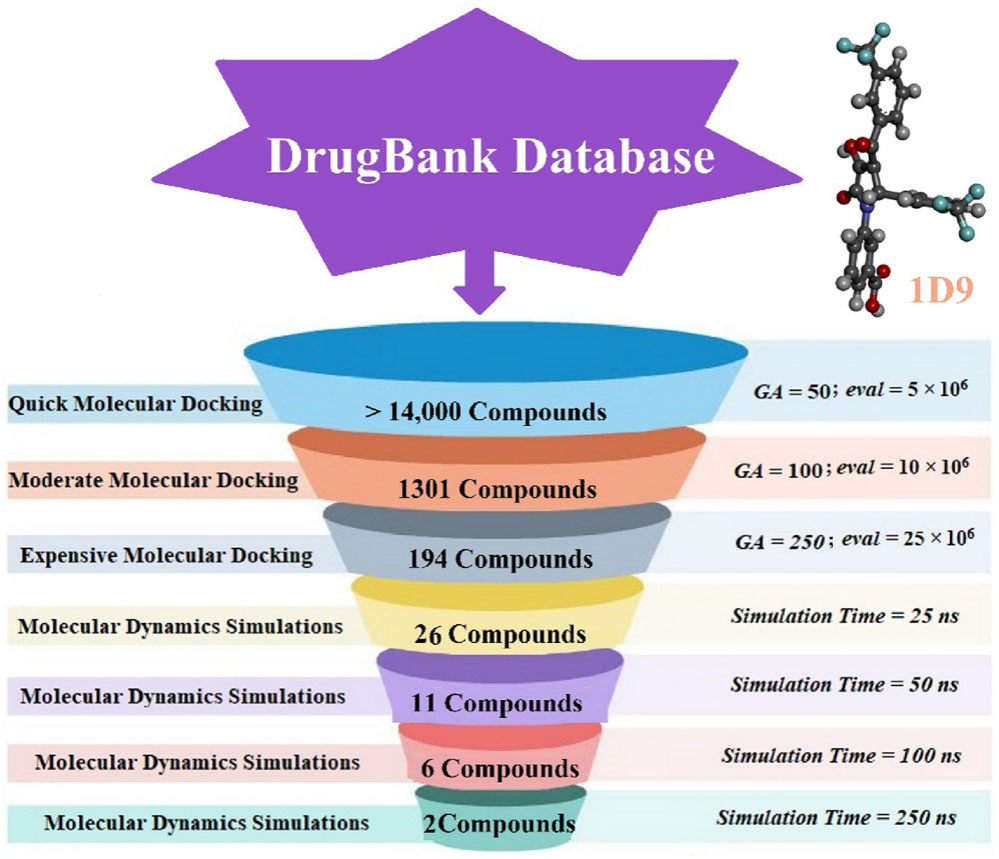
Schematic workflow of the employed in silico techniques to filtrate the DrugBank database against VP35.

Given the lengthy durations necessary for drug development, repurposing current drugs with protective effects against EBOV offers a more rapid development approach.^[^ ^20^ ^]^ A significant benefit of drug repurposing is that the safety profiles of these medications are already established, making it probable that a sufficient stock of drugs is accessible for prompt utilization. Recent studies have underscored the utility of drug repurposing frameworks in accelerating the identification of antiviral, anticancer, and antiparkinson agents.^[^ 21, 22, 23 ^]^ Shamsi et al. investigated the potential of repurposing FDA‐approved drugs using integrative in silico approaches to accelerate anticancer and antiparkinson drug discovery.^[^ ^24^ ^,^ ^25^ ^]^ Currently, ongoing research concentrates on evaluating the effectiveness of several repurposed medications to fight EBOV.^[^ 26, 27, 28 ^]^ However, the putative role of the DrugBank database as a source of VP35 inhibitors has not yet been evaluated extensively. Herein, the DrugBank database, containing > 14000 drug candidates, was explored to identify the most potent VP35 inhibitors utilizing various in silico approaches. Such approaches, including docking computations and molecular dynamics simulations (MDS), were employed to obtain energetic and structural insights into VP35 inhibition. Notably, MDS was conducted over 250 ns in triplicate to guarantee the accuracy of the findings, followed by binding affinity computation utilizing the molecular mechanics/generalized Born surface area (MM/GBSA) approach. Upon the MM/GBSA//250 ns MDS, post‐MD analyses were investigated for the most promising candidates complexed with VP35. Ultimately, the molecular reactivity of the identified candidates was inspected by the density functional theory (DFT) computations. The schematic workflow of the employed in silico techniques to filtrate the DrugBank database against VP35 is illustrated in **Figure** **1**. These findings may open avenues for future research to explore unprecedented ways to inhibit VP35. Of note, computational studies simplify biological systems and cannot fully replicate the complexity of living organisms, including factors such as cellular uptake, metabolism, off‐target effects, and immune responses. The main limitation of this study is the absence of experimental confirmation for the identified VP35 inhibitors, highlighting the need for subsequent in vitro and in vivo evaluations to offer a deeper understanding of the therapeutic potential of the identified candidates to fight EBOV infection.

## 2 Results and Discussion

### 2.1 Docking Validation

**Figure 2:**
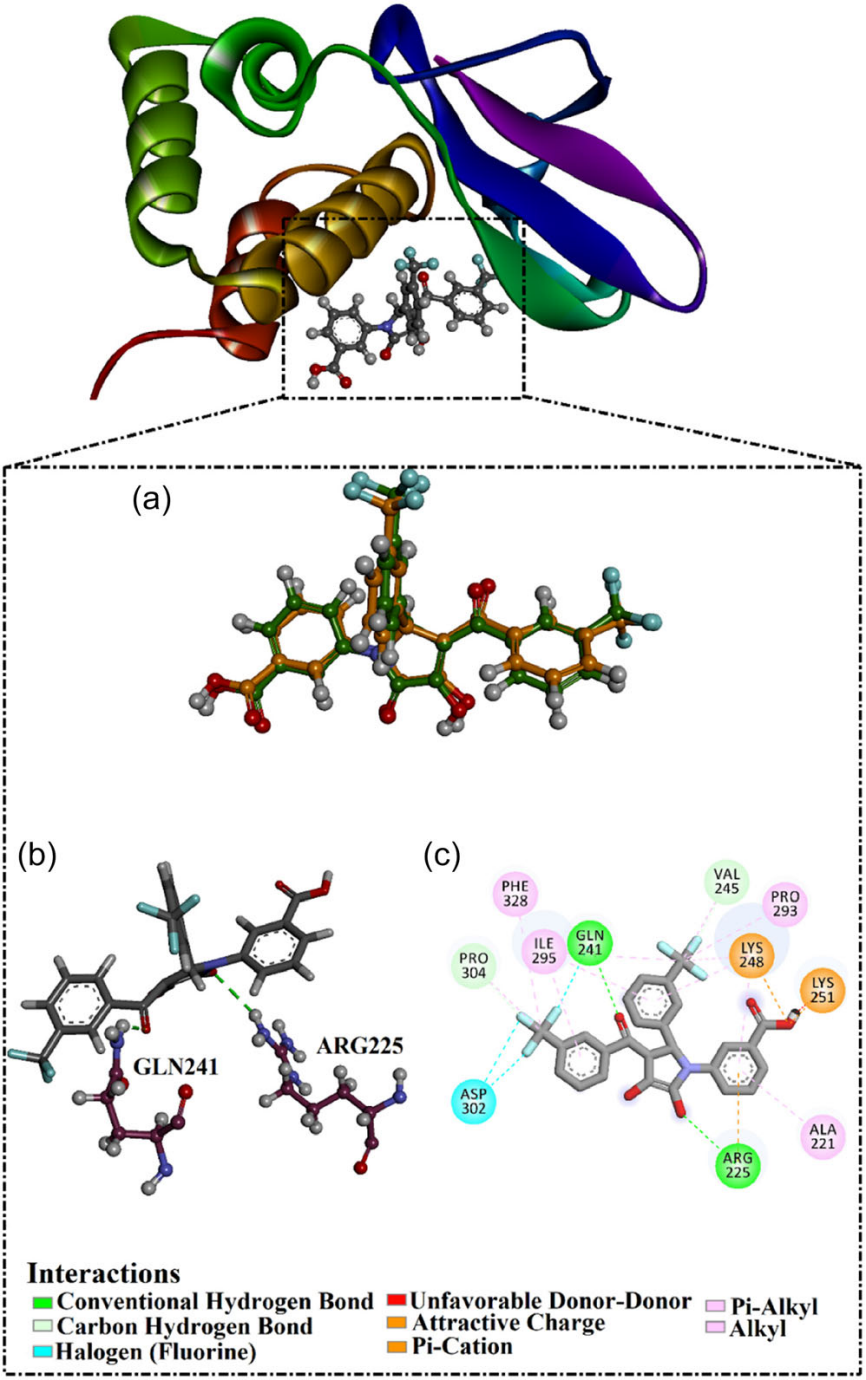
a) 3D superimposition of the predicted (orange) and experimental (green) binding modes, and b) 3D and c) 2D representations of the predicted docking pose of 1D9 toward VP35.

To assess the employed docking protocol, redocking of the cocrystallized 1D9 inhibitor was executed toward VP35 (PDB ID: 4IBJ^[^ ^29^ ^]^). It has been reported that the root‐mean‐square deviation (RMSD) between the native binding mode and the predicted docking pose should be less than 2.0 Å.^[^ 30, 31, 32 ^]^ The calculated RMSD between the native binding mode and the anticipated docking pose was found to be 0.85 Å, demonstrating that the two poses almost entirely overlapped (**Figure** **2**). These results unveiled that AutoDock4.2.6 software could minutely and reasonably anticipate the correct binding pose of the ligand within the VP35 active site. Furthermore, the employed docking protocol was trustworthy for mining the DrugBank database to hunt putative VP35 inhibitors. Inspecting the docking pose of 1D9 within the VP35 active site demonstrated that the fluorine atom and the OH of 1D9 established two H‐bonds with the NH_3_ of ARG225 (2.63 Å) and NH_2_ of GLN241 (1.80 Å). Finally, the predicted docking outcomes of 1D9 within the VP35 active site displayed a good docking score of −6.4 kcal mol^−1^.

### 2.2 DrugBank Database Screening

To minimize computational expenses and processing time, the DrugBank database, containing > 14000 drug candidates, was mined to identify potent VP35 inhibitors utilizing quick docking parameters (i.e., *eval* = 5 × 10^6^ and *GA* = 50). From quick docking computations, only 1301 drug candidates revealed docking scores lower than 1D9 (ligand control, calc. −6.4 kcal mol^−1^). These drug candidates were selected and underwent moderate docking estimations with *eval* = 10 × 10^6^ and *GA* = 100. Besides, the corresponding docking scores of these drug candidates are gathered in Table S1, Supporting Information. From Table S1, Supporting Information, only 194 drug candidates unveiled docking scores ≤ −8.0 kcal mol^−1^. As a consequence, these candidates were submitted to expensive docking calculations with *eval* = 25 × 10^6^ and *GA* = 250. Table S2, Supporting Information, lists the predicted docking scores of these 194 drug candidates toward VP35. According to the data provided in Table S2, Supporting Information, only 26 drug candidates demonstrated docking scores of ≤ −9.0 kcal mol^−1^. It is worth noting that −8.0 and −9.0 kcal mol^−1^ were chosen as cutoff points to shortlist the most promising VP35 inhibitors. Figure S1, Supporting Information, depicts the 2D representations of the binding patterns for these drug candidates with the essential binding residues of VP35. As depicted in Figure S1, Supporting Information, most of the inspected drug candidates exhibited similar docking poses, forming a fundamental H‐bond with GLN241 and other H‐bonds with various residues within the active site of VP35. Other interactions, including van der Waals, π‐based, and hydrophobic interactions, were also noticed between the investigated drug candidates and VP35, resulting in high binding affinities (Figure S1, Supporting Information). The 2D chemical structures and evaluated docking scores for the top two potent drug candidates are presented in **Table** **1**. Of note, these two drug candidates were selected according to the estimated MM/GBSA binding energy over 250 ns MDS outlined below.

**Table 1:** 2D chemical structures and evaluated quick, moderate, and expensive docking scores of the most promising two drug candidates against VP35.

No.a)	Compound name/ID	2D chemical structure	Docking score [kcal mol^−1^]		
			Quick	Moderate	Expensive
	1D9	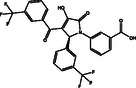	−6.5	−6.4	−6.4
1	DB14875 (AZD‐4017)	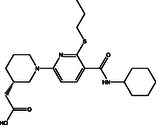	−9.7	−10.3	−10.7
2	DB07800 (*N*‐(2‐(((5‐chloro‐2‐pyridinyl)amino)sulfonyl)phenyl)‐4‐(2‐oxo‐1(2H)‐pyridinyl)benzamide)	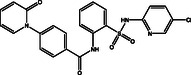	−9.6	−10.2	−10.6

a) Data was arranged based on the expensive docking scores.

**Figure 3:**
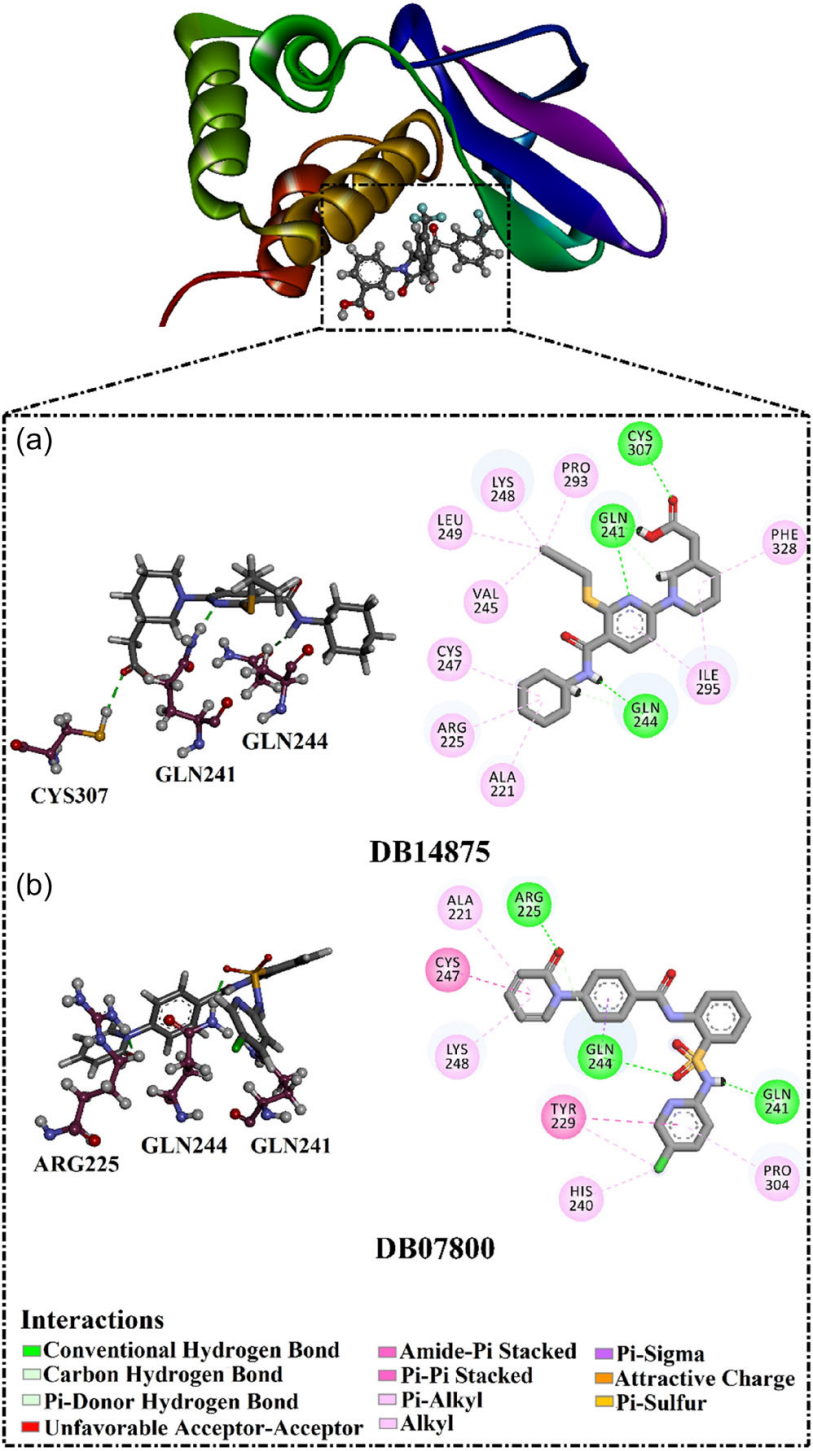
3D and 2D illustrations of the predicted docking poses of a) DB14875 and b) DB07800 within the VP35 active site.

DB14875 is a selective and potent inhibitor toward 11β‐hydroxysteroid dehydrogenase type 1 (11β‐HSD1), demonstrating a promising IC_50_ with a value of 7.0 nM.^[^ ^33^ ^]^ DB14875 showed the lowest docking score of −10.7 kcal mol^−1^ toward VP35 (Table 1). Inspecting the docking pose of DB14875 within VP35 indicated that NH of *N*‐methyl formamide exhibited an H‐bond with CO of GLN244 (2.32 Å) (**Figure** **3**). Besides, carboxylic acid formed two H‐bonds with CO of GLN241 (1.80 Å) and SH of CYS307 (2.35 Å). The N atom of pyridine exhibited an H‐bond with NH_2_ of GLN241 (1.78 Å). As well, DB14875 displayed carbon H‐bonds with GLN244 and GLN241 residues.

DB07800, an orally bioavailable fXa inhibitor,^[^ ^34^ ^]^ also showed a good docking score (calc. −10.6 kcal mol^−1^). Examining the docking pose of DB07800 within the VP35 active site revealed that the two NH groups exhibited two H‐bonds with CO of GLN241 (2.08 Å) and NH_2_ of ARG225 (1.99 Å) (Figure 3). Additionally, the oxygen atom of sulfur dioxide interacted by the formation of an H‐bond with NH_2_ of GLN244 (1.88 Å). DB07800 also exhibited π–π stacked interaction with TYR229, π‐sigma interaction with GLN244, and amide‐π stacked interaction with CYS307. Ultimately, DB07800 established carbon H‐bonds with ARG225 and GLN244 residues.

### 2.3 Molecular Dynamics Simulations (MDS)

**Figure 4:**
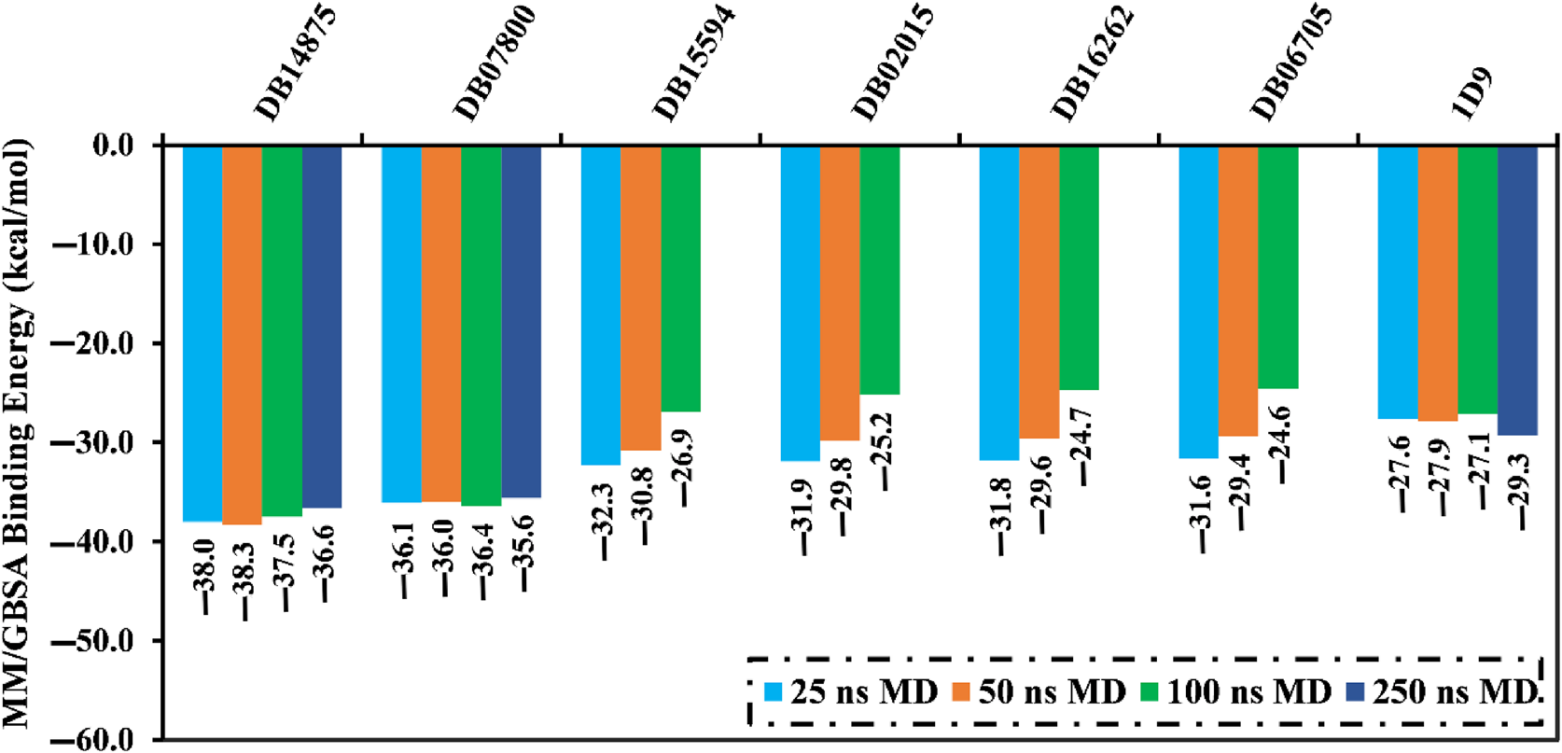
Evaluated binding affinities (in kcal mol^−1^) over 25, 50,100, and 250 ns MDS for the top potent drug candidates toward VP35.

MDS has become an indispensable tool in the field of structural biology and drug discovery. MDS offers a comprehensive and dynamic view of protein‐inhibitor complexes, enabling a deeper comprehension of their interactions, stability, and functional effects.^[^ ^35^ ^,^ ^36^ ^]^ This knowledge is invaluable for guiding experimental studies and advancing therapeutic development.^[^ ^37^ ^,^ ^38^ ^]^ Therefore, the most potent drug candidates (26 drug candidates with docking scores < −9.0 kcal mol^−1^) were subjected to MDS over 25 ns, accompanied by binding affinities estimation. The computed MM/GBSA binding energies over 25 ns are compiled in Table S3, Supporting Information. As per Table S3, Supporting Information, only 11 drug candidates showed greater binding affinities (Δ*G*_binding_) compared to 1D9 (calc. −27.6 kcal mol^−1^). To obtain more reliable binding affinities of the investigated drug candidates complexed with VP35, these drug candidates were further subjected to 50 ns MDS, and their MM/GBSA binding energies were estimated (Table S4, Supporting Information). From the recorded data in Table S4, Supporting Information, only six drug candidates unveiled binding affinities higher than 1D9 (Δ*G*_binding_ = −27.9 kcal mol^−1^). Consequently, these six drug candidates were nominated for 100 ns MDS, pursued by binding affinity evaluations (**Figure** **4**). Based on the data presented in Figure 4, only two drug candidates—namely DB14875 and DB07800—revealed binding energies lower than 1D9 (Δ*G*_binding_ = −27.1 kcal mol^−1^). The evaluated Δ*G*_binding_ for DB14875 and DB07800 complexed with VP35 over 100 ns MDS were −37.5 and −36.4 kcal mol^−1^, respectively. For these two promising drug candidates complexed with VP35, the MDS was extended to 250 ns, accompanied by binding energy calculations. Remarkably, there is no significant difference in the evaluated binding affinities throughout the 100 and 250 ns MDS for DB14875‐ and DB07800‐VP35 complexes. In comparison to the binding energy of 1D9 (calc. −29.3 kcal mol^−1^), DB14875 and DB07800 disclosed lower binding energies towards VP35 over the 250 ns MDS, with average Δ*G*_binding_ values of −36.6 and −35.6 kcal mol^−1^, respectively. In order to obtain more dependable results, MDS was executed over 250 ns in triplicate for DB14875 and DB07800 bound to VP35, accompanied by binding energy calculations (Table S5, Supporting Information). Based on the data listed in Table S5, Supporting Information, the investigated drug candidates revealed approximately identical binding energies in triplicate. For instance, DB14875 demonstrated average Δ*G*_binding_ values of −36.6, −36.2, and −36.5 kcal mol^−1^ in the triplicates. These findings indicated that DB14875 and DB07800 are promising VP35 inhibitors and might be rational drug candidates for EBOV therapy.

**Figure 5:**
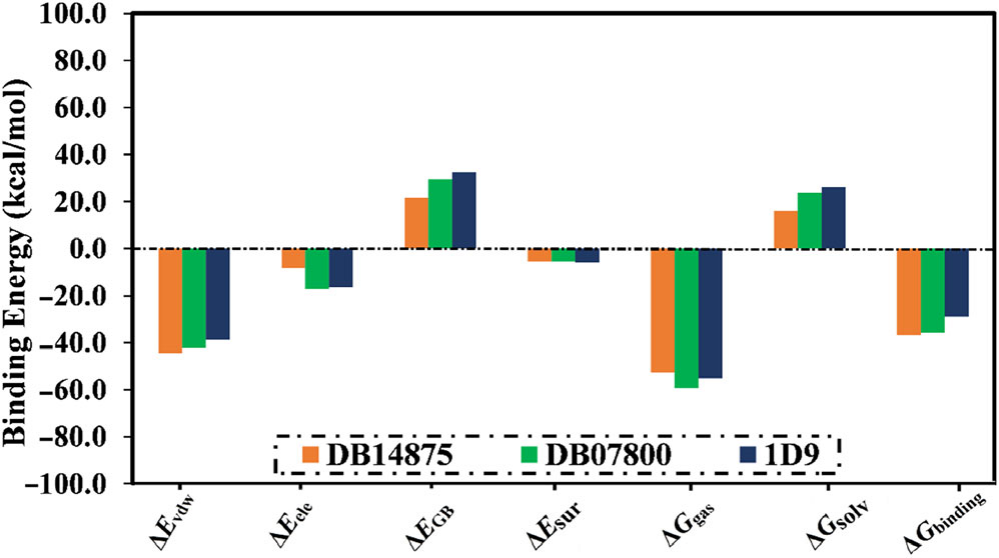
Binding energy components of DB14875, DB07800, and 1D9 complexed with VP35 over 250 ns MDS.

The binding energy decomposition was carried out to highlight the nature of the interactions between the identified drug candidates and VP35 (**Figure** **5**). According to data presented in Figure 5, the *E*_vdW_ was the primary participant in the binding energies of DB14875, DB07800, and 1D9, with average values of −44.5, −42.2, and −38.7 kcal mol^−1^, respectively. Additionally, the *E*_ele_ was appropriate for DB14875‐, DB07800‐, and 1D9‐VP35 complexes with average values of −8.3, −17.3, and −16.5 kcal mol^−1^, respectively.

**Figure 6:**
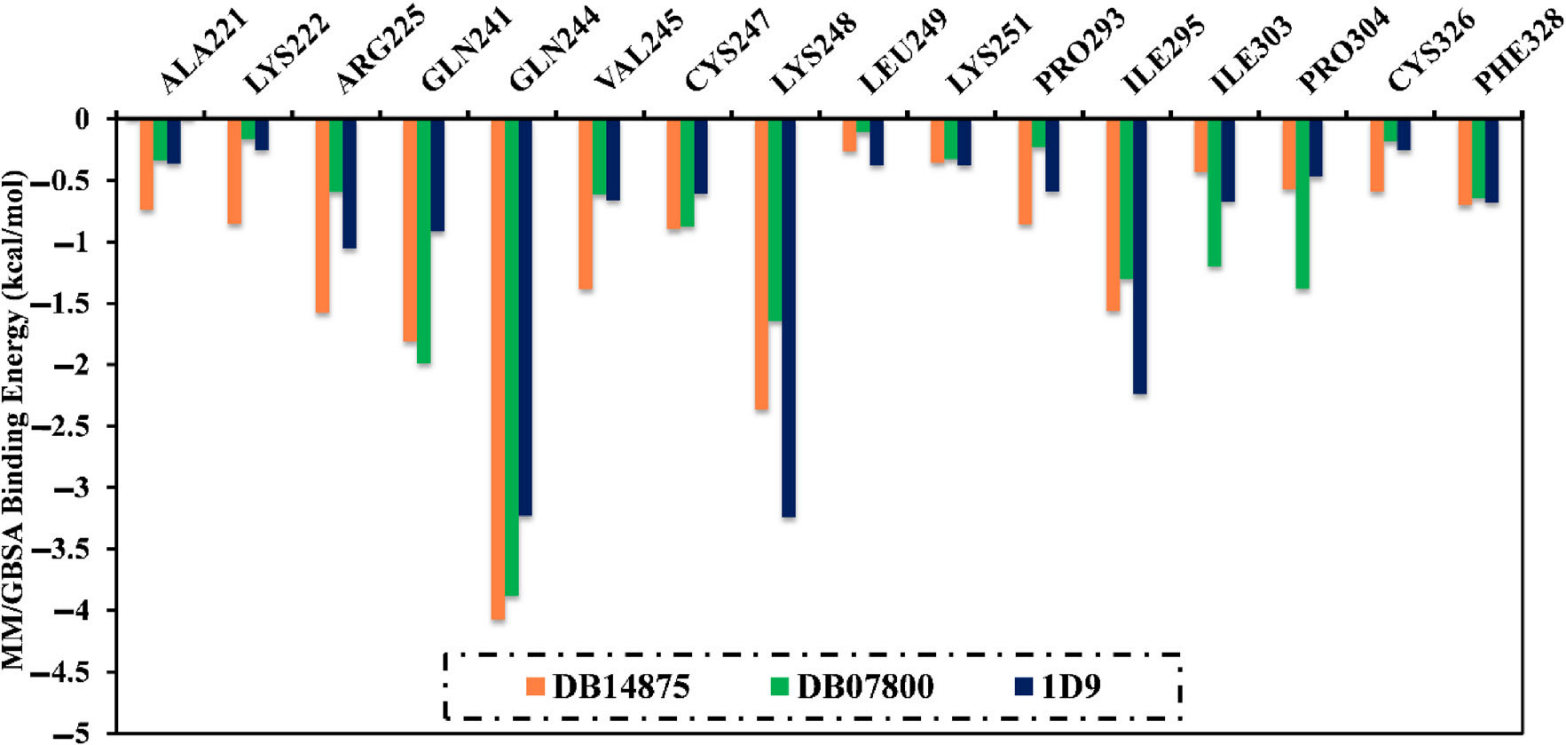
Per‐residue energy decomposition analysis of the identified drug candidates and 1D9 complexed with VP35 over 250 ns MDS.

The per‐residue energy decomposition analysis was performed to examine the interactions of DB14875, DB07800, and 1D9 with the crucial amino acids of the VP35 active site (**Figure** **6**). Only amino acids with Δ*G*_binding_ < −0.5 kcal mol^−1^ were considered. As evident in Figure 6, GLN244, GLN241, LYS248, and ARG225 demonstrated promising participation in the binding of DB14875, DB07800, and 1D9 with VP35. For instance, GLN244 exhibited binding energy with Δ*G*_binding_ values of −4.1, −3.9, and −3.2 kcal mol^−1^ for DB14875‐, DB07800‐, and 1D9‐VP35 complexes, respectively.

### 2.4 Post‐MD Analyses

To evaluate the steadiness of DB14875, DB07800, and 1D9 complexed with VP35, a series of post‐MD analyses were performed over 250 ns MDS. These analyses involved binding energy per‐frame, radius of gyration (Rg), root–mean‐square fluctuation and deviation (RMSF and RMSD), H‐bond analysis, and center‐of‐mass (CoM) distance.

#### 2.4.1 Binding Energy Per‐Frame

**Figure 7:**
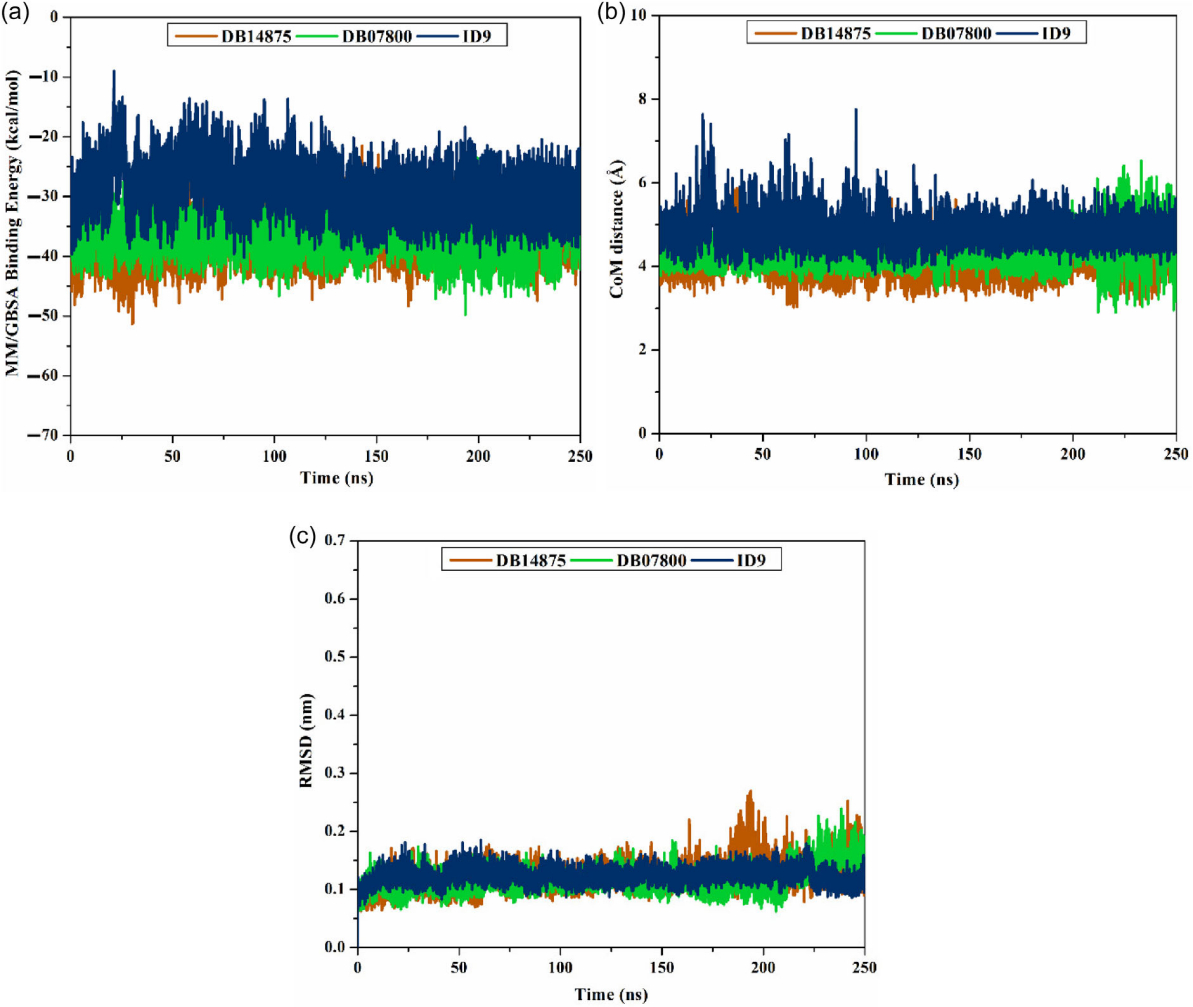
a) Binding energy per‐frame, b) CoM distance, and c) RMSD for the backbone, relative to the initial coordinates of DB14875 (orange), DB07800 (green), and 1D9 (blue) against VP35 over the 250 ns MDS.

The energetic stability of DB14875‐, DB07800‐, and 1D9‐VP35 complexes was thoroughly assessed over 250 ns MDS by examining the correlation between binding energy and time (**Figure** **7**a). Notably, complete stability was observed for DB14875‐, DB07800‐, and 1D9‐VP35 complexes, with average Δ*G*_binding_ values of −36.6, −35.6, and −29.3 kcal mol^−1^, respectively. These findings revealed that all the investigated drug‐VP35 complexes preserved constancy over the 250 ns MDS.

#### 2.4.2 CoM Distance

The CoM distance of the identified drug candidates and GLN244 was gauged to gain a deeper understanding of the steadiness of the drug‐VP35 complexes over the 250 ns MDS (Figure 7b). As shown in Figure 7b, the CoM distance remained consistent for DB14875‐, DB07800‐, and 1D9‐VP35 complexes with average values of 4.1, 4.3, and 4.9 Å, respectively. This analysis confirmed the robust stability of the investigated drug candidates bound to VP35 throughout the 250 ns MDS.

#### 2.4.3 RMSD Analysis

To clarify the conformational alterations of the identified drug candidates bound to VP35, the RMSD of the backbone atoms from the simulation frames was estimated (Figure 7c). Notably, the RMSD values for DB14875‐, DB07800‐, and 1D9‐VP35 complexes stayed beneath 0.2 nm throughout the 250 ns MDS. These results highlighted that the identified drug candidates are firmly anchored in the active site and do not disrupt the overall structure of VP35.

#### 2.4.4 H‐Bond Analysis

**Figure 8:**
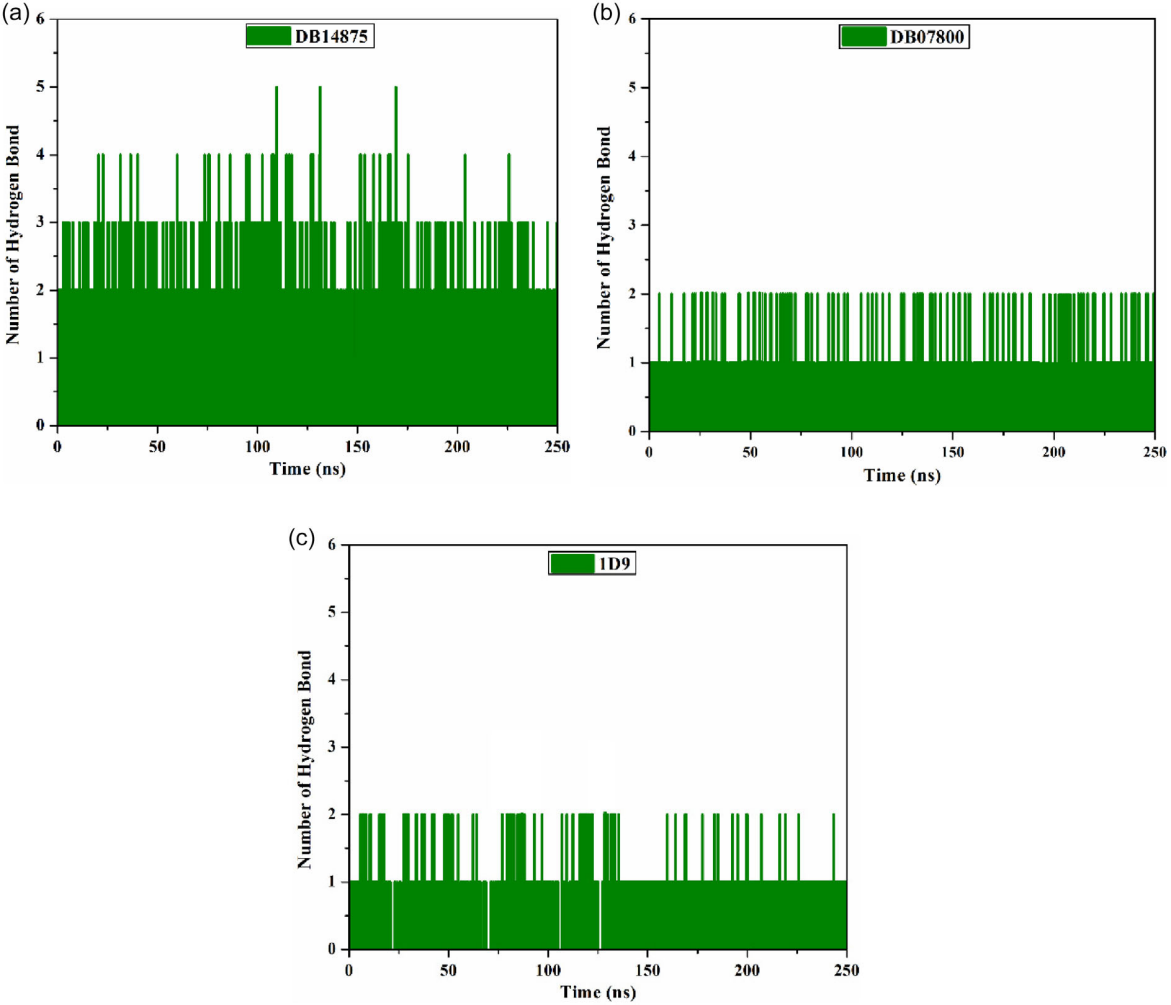
Number of H‐bonds for a) DB14875, b) DB07800, and c) 1D9 in complex with VP35 over 250 ns MDS.

Moreover, the stabilization of DB14875, DB07800, and 1D9 complexed with VP35 was assessed by measuring the H‐bond number over 250 ns MDS. **Figure** **8** illustrates the correlation between the H‐bond number and simulation time. As depicted in Figure 8, the average H‐bond number was 3, 2, and 1 for DB14875‐, DB07800‐, and 1D9‐VP35 complexes over 250 ns MDS. Remarkably, 1D9 exhibited the least H‐bond number with the key residues within the active site of VP35. Nevertheless, the strong binding affinity of 1D9, with an average Δ*G*_binding_ of −29.3 kcal mol^−1^, may be attributed to other types of interactions, including vdW, hydrophobic, and *π*‐based interactions. These findings indicated that the DB14875‐and DB07800‐VP35 complexes are significantly more stable than those of 1D9.

#### 2.4.5 RMSF Analysis

**Figure 9:**
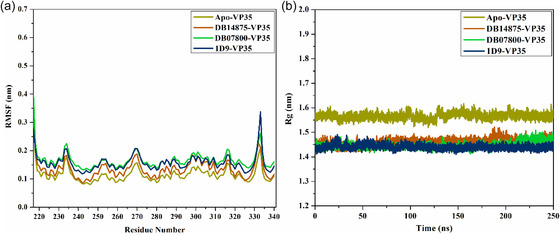
a) RMSF and b) Rg of apo‐VP35 (dark yellow), DB14875‐VP35 (orange), DB07800‐VP35 (green), and 1D9‐VP35 (blue) over 250 ns MDS.

The RMSF was evaluated in relation to the backbone atom of each residue, and the RMSF graph was utilized to illustrate the fluctuations occurring at the residue level over 250 ns MDS (**Figure** **9**a). As illustrated in Figure 9a, the RMSF plot indicated that the binding of DB14875, DB07800, and 1D9 to VP35 was steady and had no major impact on the flexibility of VP35 over the 250 ns MDS. The average RMSF values for apo‐, DB14875‐, DB07800‐, and 1D9‐VP35 were 0.11, 0.13, 0.16, and 0.15 nm, respectively.

#### 2.4.6 Rg Analysis

Furthermore, the Rg analysis was accomplished to estimate the compactness and overall steadiness of the investigated complex based on ligand binding throughout 250 ns MDS (Figure 9b). The Rg is defined by taking the root–mean‐square of the distances from every atom in the molecule to its center of mass. The values derived from the trajectory for Rg are inversely related to the distance from its axis of rotation. A higher Rg value indicates that the protein folding is less dense and more loosely arranged, suggesting that the compounds are situated further away from the center of mass. The average Rg values for apo‐, DB14875‐, DB07800‐, and 1D9‐VP35 were 1.47, 1.46, 1.45, and 1.44 nm, respectively. Ultimately, these findings proved that the complexation of VP35 with the identified drug candidates increased the compactness of VP35, which results in enhanced overall stability.

### 2.5 Quantum Mechanical (QM) Calculations

**Figure 10:**
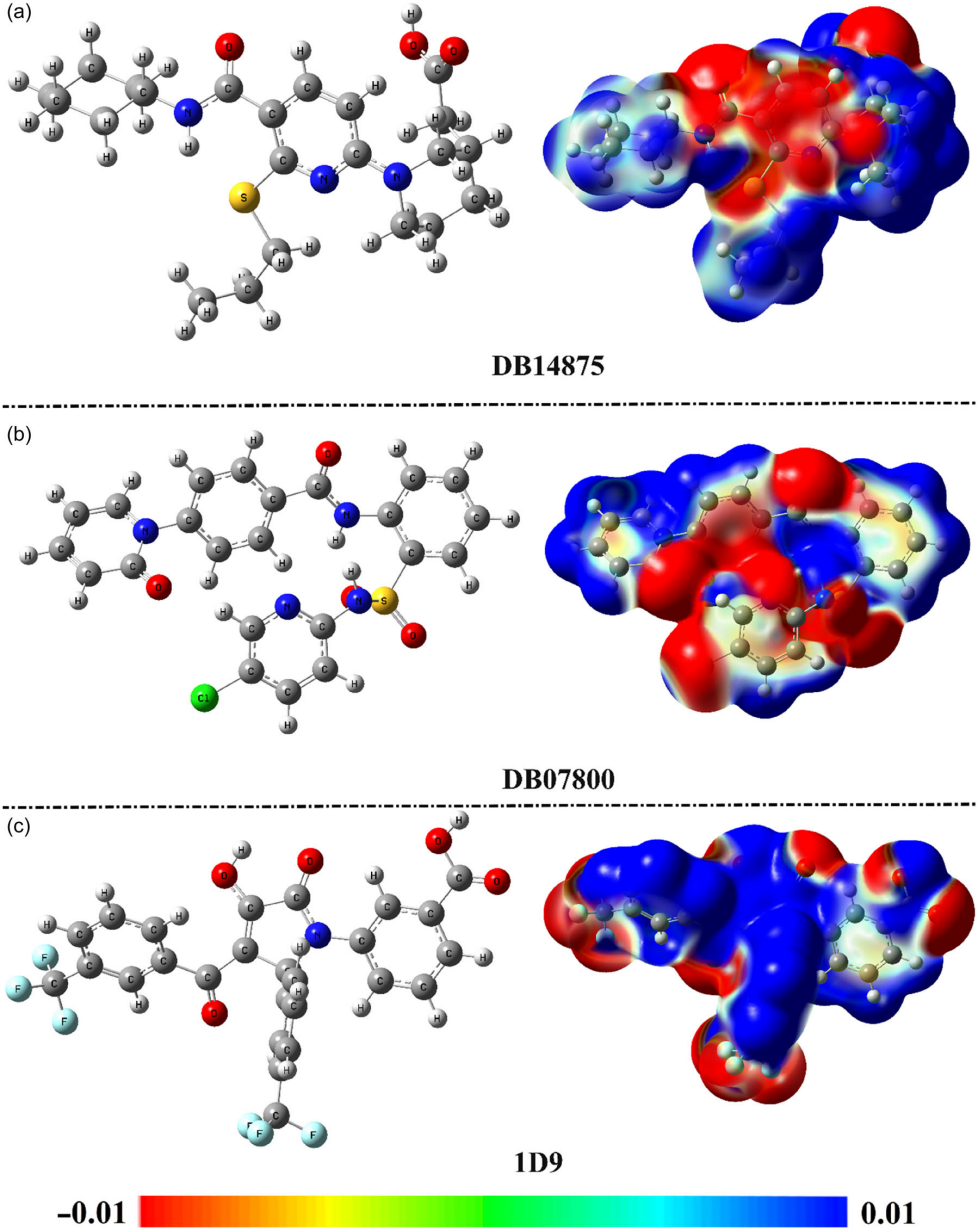
Optimized structures and MEP maps for a) DB14875, b) DB07800, and c) 1D9.

The electrostatic potential (ESP) surface is a method used to describe a molecular entity by highlighting the areas that are electrophilic and nucleophilic. The ESP surface depicts electropositive regions in blue and electronegative areas in red. Upon the optimized structures of DB14875, DB07800, and 1D9, the molecular electrostatic potential (MEP) maps were generated and plotted (**Figure** **10**).

By analyzing the MEP maps, red sites were found around O and N atoms, while blue color regions were noticed around H atoms. Of note, the investigated drug candidates revealed the capability of establishing H‐bonds with the fundamental residues within the VP35 active site.

**Figure 11:**
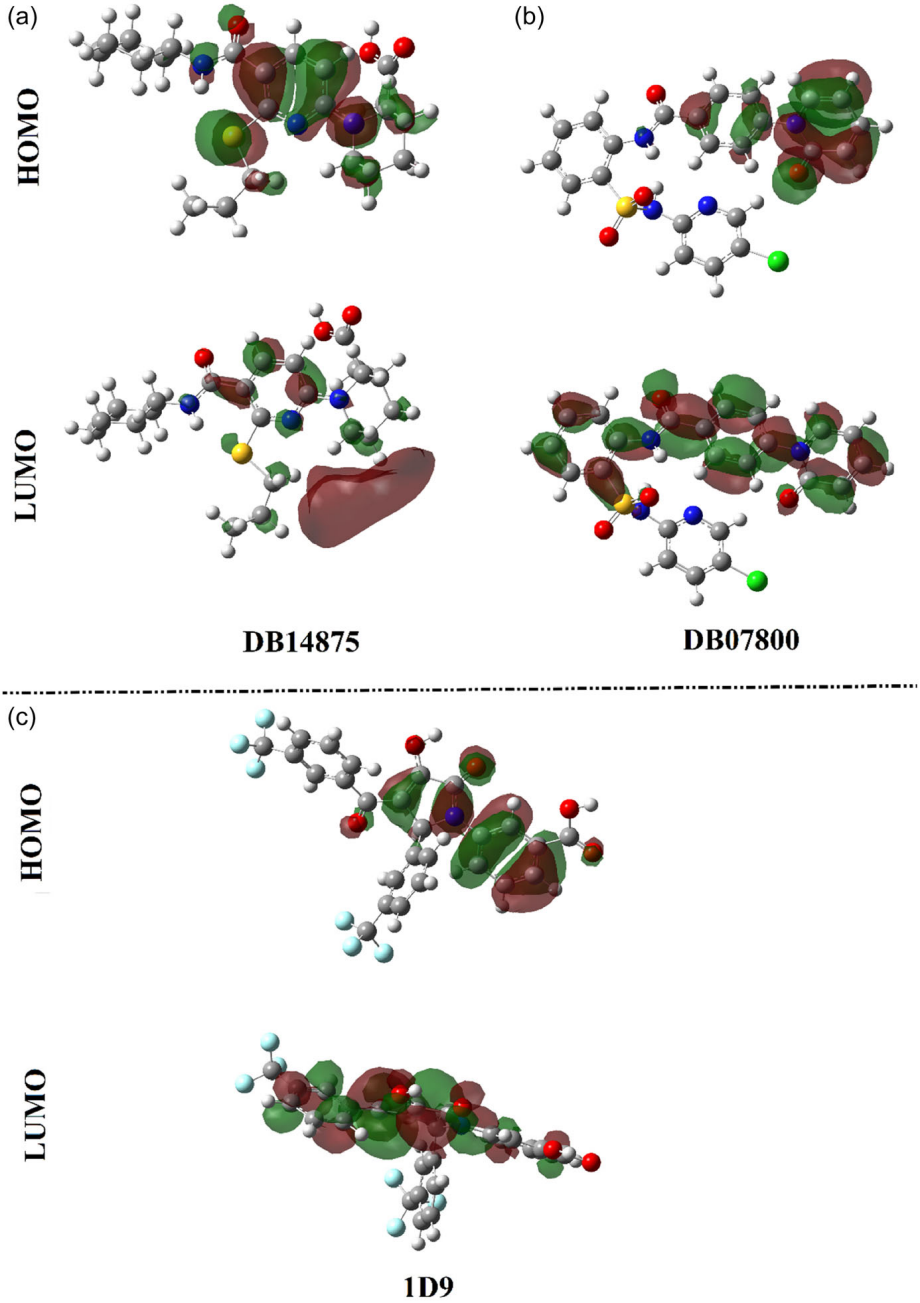
HOMO and LUMO distribution plots for the optimized a) DB14875, b) DB07800, and c) 1D9.

From an electronic perspective, frontier molecular orbital (FMO) theory was applied to all investigated drug candidates. In this context, the highest occupied molecular orbital (HOMO) and lowest unoccupied molecular orbitals (LUMO) levels were mapped for DB14875, DB07800, and 1D9 (**Figure** **11**). The values of *E*_HOMO_, *E*_LUMO_, and *E*_gap_ were computed and are compiled in **Table** **2**.

**Table 2:** Evaluated electronic parameters and quantum chemical descriptors for the identified drug candidates and 1D9.

Compound name/code	*E*_HOMO_ [eV]	*E*_LUMO_ [eV]	*E*_gap_ [ev]	*IP* [eV]	*EA* [eV]	*η* [eV]	*S* [eV^−1^]
1D9	−8.35	−2.09	6.26	8.35	2.09	3.13	0.32
DB14875	−6.85	−0.09	6.76	6.85	0.09	3.38	0.30
DB07800	−7.73	−1.28	6.45	7.73	1.28	3.22	0.31

From Figure 11, the HOMO levels were primarily located around the nucleophilic sites, specifically the O and N atoms. In contrast, the LUMO levels were predominantly found in the electrophilic areas, particularly the H atoms of the investigated drug candidates and 1D9.

As listed in Table 2, the *E*_HOMO_/*E*_LUMO_ values for DB14875, DB07800, and 1D9 were −6.85/–0.09, −7.73/−1.28, and −8.35/−2.09 eV, respectively. As well, the investigated drug candidates and 1D9 demonstrated low *E*_gap_ values ranging from 6.26 to 6.76 eV, demonstrating their considerable chemical reactivity.

Quantum chemical descriptors play a crucial role in understanding the chemical reactivity of the investigated drug candidates. For the identified drug candidates and 1D9, different reactivity indices were computed and are gathered in Table 2. According to Table 2, the *IP* values for DB14875, DB07800, and 1D9 were 6.85, 7.73, and 8.35 eV, respectively. Additionally, the DB14875, DB07800, and 1D9 demonstrated electron affinity (*EA*) with values of 0.09, 1.28, and 2.09 eV, respectively. The hardness (*η*) and softness (*S*) of the optimized drug candidates and 1D9 served as indicators of their chemical reactivity. The *η* values for DB14875, DB07800, and 1D9 were 3.38, 3.22, and 3.13 eV, respectively. DB14875, DB07800, and 1D9 displayed good *S* with values of 0.30, 0.31, and 0.32 eV^–1^, respectively, suggesting a great capacity for electronic adability. The calculated *η* and *S* values for DB14875, DB07800, and 1D9 demonstrated favorable polarizability and a balanced ability to donate and accept electrons, which supported strong noncovalent interactions, such as H‐bond, electrostatic, and π–π stacking, with the binding pocket of VP35.

## 3 Conclusion

EBOV causes hemorrhagic fever syndrome, which remains a major health concern worldwide. The elevated mortality rate linked to EBOV highlights the pressing necessity to create pharmaceuticals that can obstruct various phases of the EBOV replication process. The VP35 impedes the production of host IFN‐α/β by interfering with the immune system's responses to viral infection, making it a charming druggable target for identifying drug candidates to combat EBOV infection. Herein, the DrugBank database, including > 14000 drug candidates, was virtually screened to hunt prospective VP35 inhibitors utilizing docking computations. The most promising candidates, with expensive docking scores < −9.0, were submitted to MDS accompanied by MM/GBSA binding energy evaluations. Upon the MM/GBSA binding energy results over 250 ns MDS, DB14875 and DB07800 demonstrated superior binding affinity against VP35 compared to 1D9 with Δ*G*_binding_ values of −36.6, −35.6, and −29.3 kcal mol^−1^, respectively. The post‐MD analyses assured the identified drug candidates’ steadiness throughout 250 ns MDS. DFT computations were also executed, and their results revealed favorable molecular reactivity and stability of the identified drug candidates. The results suggested that the identified drug candidates could serve as effective inhibitors of VP35, indicating their potency to be developed into anti‐EBOV medications following further validation and testing through in vitro/in vivo assays.

## 4 Computational Methodology

### 4.1 VP35 Preparation

The crystal structure of VP35 complexed with 1D9 (PDB ID: 4IBJ^[^ ^29^ ^]^) was retrieved from the RCSB website.^[^ ^39^ ^]^ The utilized PDB was selected based on its high resolution (1.54 Å) and complete amino acid sequence. For preparation purposes, all heteroatoms involving water molecules, ligand, and ions were removed. Subsequently, the PROPKA program was used to determine the ionization states of titratable residues, followed by the addition of the missing hydrogen atoms.^[^ ^40^ ^]^

### 4.2 DrugBank Preparation

In the current research, the drug repurposing strategy was conducted on the DrugBank database, containing > 14000 investigational and approved drugs.^[^ ^41^ ^,^ ^42^ ^]^ All drug candidates of the DrugBank database were first saved in an SDF format file. According to the International Chemical Identifier (InChIKey), the redundant candidates were eliminated.^[^ ^43^ ^]^ Omega2 software was applied to generate the 3D conformations of the inspected drug candidates.^[^ ^44^ ^,^ ^45^ ^]^ Thereafter, the generated 3D structures underwent energy minimization utilizing the MMFF94S force field in SZYBKI software.^[^ ^46^ ^,^ ^47^ ^]^ The protonation states of the investigated drug candidates were determined utilizing the Fixpka program implemented inside the QUACPAC package.^[^ ^48^ ^]^ The Gasteiger‐Marsili method was employed to calculate the atomic charges of inspected drug candidates.^[^ ^49^ ^]^ Prepared repurposed drugs can be accessed at www.compchem.net/ccdb.

### 4.3 Molecular Docking

All docking evaluations were accomplished utilizing AutoDock4.2.6 software.^[^ ^50^ ^]^ In accordance with the docking protocol,^[^ ^51^ ^]^ a PDBQT file of VP35 protein was generated using MGL1.5.7 tools. To lessen computational expenses and processing time, three levels of docking computations—namely quick, moderate, and expensive docking evaluations—were performed in this work, which featured 50, 100, and 250 genetic algorithm (*GA*) runs, respectively. Additionally, the maximum number of energy evaluations (*eval*) was set to 5 × 10^6^, 10 × 10^6^, and 25 × 10^6^ for quick, moderate, and expensive docking computations, respectively. The grid was configured to cover the entire binding pocket, measuring 50 × 50 × 50 Å^3^. The coordinates for the center of the grid box were located at *X* = −1.99, *Y* = 21.851, and *Z* = 22.746. The rest of the docking parameters were set to their defaults. The docking conformation with the lowest docking score was selected as a representative docking pose.

### 4.4 Molecular Dynamics Simulations (MDS)

AMBER20 software was applied to conduct MDS for top‐ranking drug candidates bound to VP35.^[^ ^52^ ^]^ More details regarding the parameters for conducting MDS are provided elsewhere.^[^ 53, 54, 55, 56 ^]^ Succinctly, VP35 was described using the AMBER force field 14SB.^[^ ^57^ ^]^ General AMBER Force Field (GAFF2) was employed to parameterize the investigated drug candidates.^[^ ^58^ ^]^ For partial atomic charge calculation, the investigated drug candidates were first optimized at the HF/6‐31G* level utilizing Gaussian09 software.^[^ ^59^ ^]^ Subsequently, the restrained electrostatic potential approach was employed to calculate the atomic charges of the optimized drug candidates.^[^ ^60^ ^]^ The electroneutrality of the investigated drug‐VP35 complex was preserved by adding sodium (Na^+^) and chloride (Cl^−^) counterions, pursued by positioning each complex within a truncated octahedral periodic box filled with TIP3P water molecules.^[^ ^61^ ^]^ Additionally, the isosmotic salt environment was conserved by inserting 0.15 M NaCl. The solvated complexes were minimized for 5000 steps using the steepest descent and conjugate gradient methods. Afterward, the minimized complexes were gently heated up to 310 K for 50 ps. Under the NPT ensemble, the heated complexes were equilibrated for 10 ns. Ultimately, the equilibrated complexes were advanced for the production stages over 25, 50, 100, and 250 ns, recording the coordinates every 10 ps for post‐MD analyses. MDS over 250 ns was carried out in triplicate on the top potent drug candidates bound to VP35. MDS was executed with pmemd.cuda GPU inside the AMBER20 software. Drug‐VP35 interactions were visualized using BIOVIA Discovery Studio Visualizer.^[^ ^62^ ^]^

### 4.5 Binding Energy Estimation

The MM/GBSA approach was employed to estimate the binding energies for VP35‐drug complexes.^[^ ^63^ ^]^ The binding energy (Δ*G*_binding_) was computed as follows

(1) $$\Delta G_{\text{binding}}=G_{\text{drug}-\mathrm{VP}35}-G_{\mathrm{VP}35}-G_{\text{drug}}$$

where the *G* is given as follows

(2) $$G=G_{\mathrm{sol}}+E_{\mathrm{MM}}-\mathrm{TS}$$

(3) $$E_{\mathrm{MM}}=E_{\mathrm{ele}}+E_{\mathrm{vdW}}+E_{\mathrm{int}}$$

(4) $$G_{\mathrm{sol}}=G_{\mathrm{SA}}+G_{\mathrm{GB}}$$

(5) $$G_{\mathrm{SA}}=0.0072\times \;\text{SASA}$$

*G*_sol_ points out the solvation‐free energy. *E*_MM_ implies molecular mechanics energy that is computed by the summation of van der Waals energy (*E*_vdW_), electrostatic energy (*E*_ele_), and internal energy (*E*_int_). *E*_int_ is the sum of angle, bond, and dihedral energies. *G*_sol_ consists of two components: the nonpolar contribution (*G*_SA_) and the polar contribution (*G*_GB_). The *G*_GB_ was calculated utilizing the amended GB model (igb = 2) proposed by Onufriev et al.^[^ ^64^ ^]^ The LCPO method was employed to determine the solvent‐accessible surface area for computing *G*_SA_.^[^ ^65^ ^]^ The entropic participation was omitted due to its high computational expense.^[^ ^66^ ^,^ ^67^ ^]^

### 4.6 QM Computations

The last frame of the identified drug candidates obtained from MDS was thoroughly inspected utilizing DFT calculations with the assistance of Gaussian09 software.^[^ ^59^ ^]^ For the investigated drug candidates, the geometrical optimization was accomplished at the M06‐2X/6‐311+G** level of theory.^[^ 68, 69, 70 ^]^ The ESP analysis utilizing an electron density envelope of 0.002 au was then performed to display the electrophilic and nucleophilic characteristics of the optimized drug candidates.^[^ ^71^ ^]^ The frontier molecular orbital (FMO) theory was applied in order to get sufficient insight into the electronic characteristics of the identified drug candidates. In this context, the HOMO/LUMO distribution patterns were mapped for the identified drug candidates. In a similar manner, the *E*_HOMO_ and *E*_LUMO_ were computed. Based on the computed *E*_LUMO_ and *E*_HOMO_, the energy gap (*E*_gap_) was computed as follows

(6) $$E_{\mathrm{gap}}=E_{\text{LUMO}}-E_{\text{HOMO}}$$

using the data derived from FMO, the electron affinity (EA)  and ionization potential (IP) were determined according to Equation ((7) and (8)), respectively.

(7) $$\text{EA}=-E_{\mathrm{LUMO}}$$

(8) $$\text{IP}=-E_{\mathrm{HOMO}}$$

The chemical reactivity of the investigated drug candidates could be evaluated according to their global reactivity descriptors. Consequently, global softness (*S*) and global hardness (*η*) were computed using Equation ((9) and (10)), respectively.

(9) $$\eta =\frac{E_{\text{LUMO}}-E_{\text{HOMO}}}{2}$$

(10) $$S=\frac{1}{\eta }$$

## Conflicts of Interest

The authors declare that there is no conflict of interests.

## Author Contributions

**Alaa H. M. Abdelrahman**: data curation (lead); formal analysis (lead); investigation (lead); visualization (lead); writing—original draft (lead), **Gamal A. H. Mekhemer**: supervision (equal); writing—review & editing (equal), **Peter A. Sidhom**: investigation (equal); visualization (equal); writing—review & editing (equal), **Mohamed A. El‐Tayeb**: methodology (equal); resources (equal); writing—review & editing (equal), **Shahzeb Khan**: project administration (equal); writing—review & editing (equal), **Mahmoud A. A. Ibrahim**: conceptualization (lead); methodology (equal); project administration (equal); resources (equal); software (lead); supervision (equal); writing—review & editing (equal).

## Supporting information

Supplementary Material

## Data Availability

The data that support the findings of this study are available in the Supporting Information Material of this paper.
