# Supplementary material for: Drug Repurposing Investigation for Combating Ebola Virus Disease: Database Mining, Docking Calculations, Molecular Dynamics, and Density Functional Theory Study
Source: ChemistryOpen. 2025 Sep 2;14(12):e202500348. doi: 10.1002/open.202500348 (PMC12680584; doi:10.1002/open.202500348)
Supplement: Supplementary file 1 — Supplementary Material [file OPEN-14-e202500348-s001.pdf]

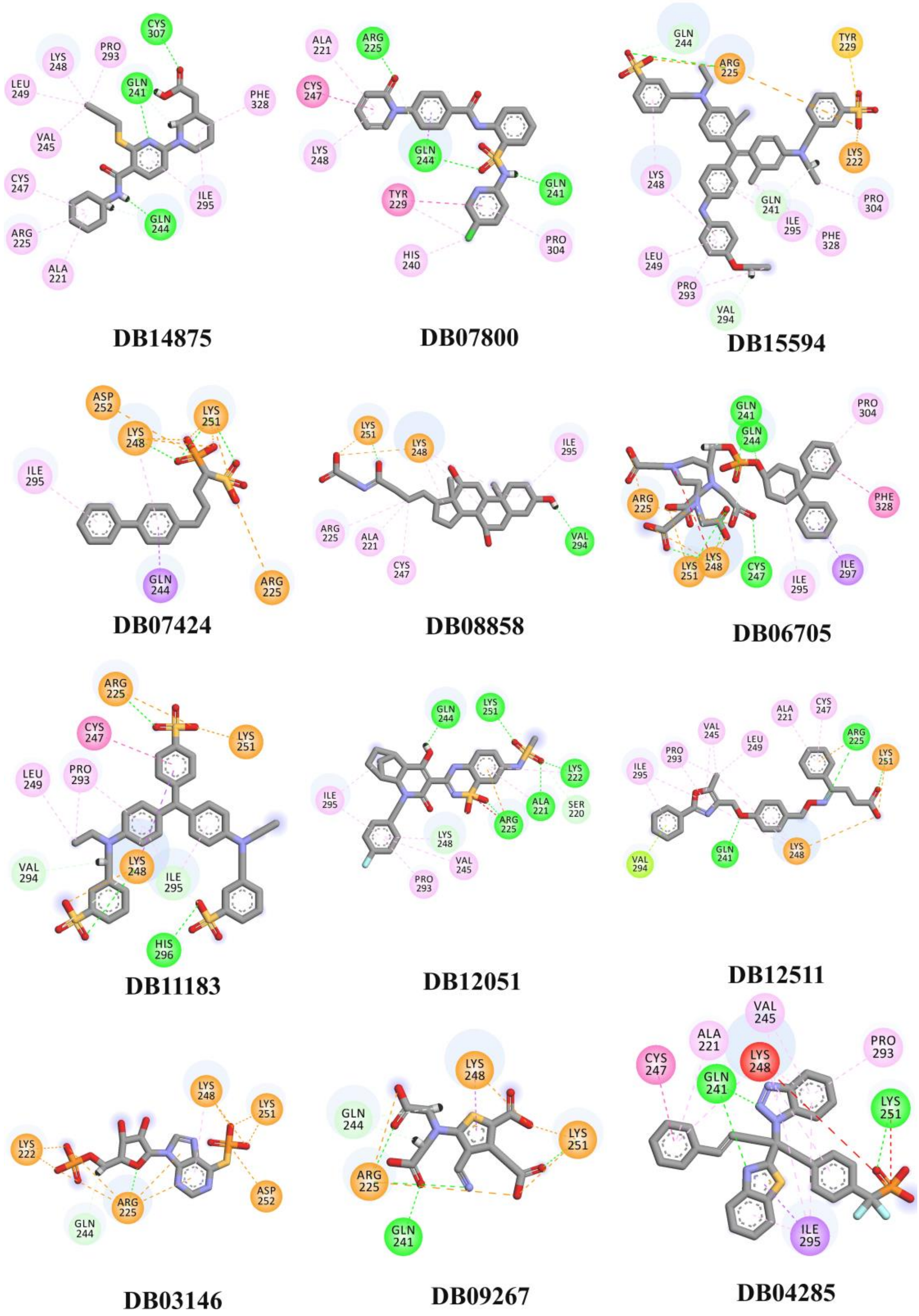

Figure S1. 2D molecular interactions of the anticipated binding modes for the top 26 drug candidates against VP35.

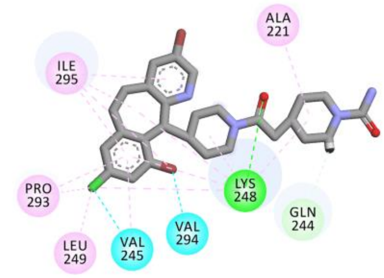

DB06448

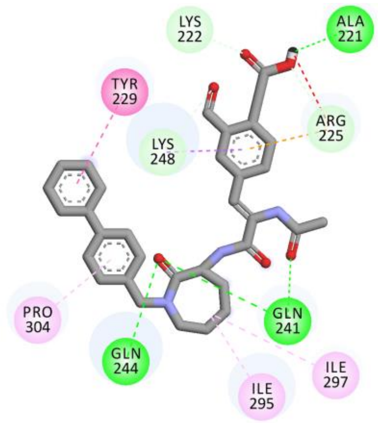

DB03104

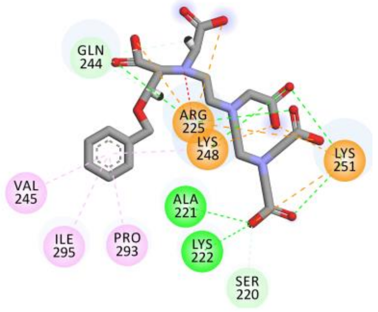

DB00743

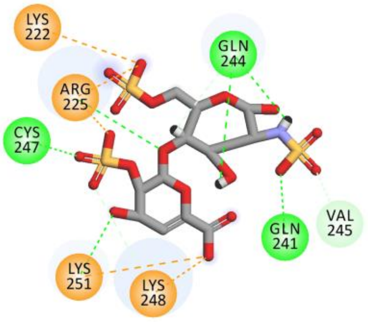

DB02322

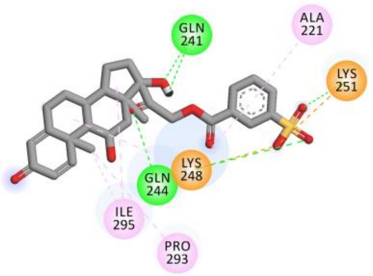

DB05340

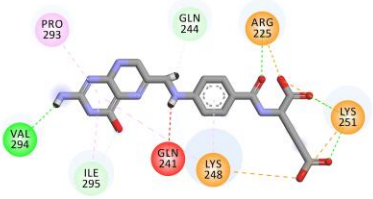

DB02015

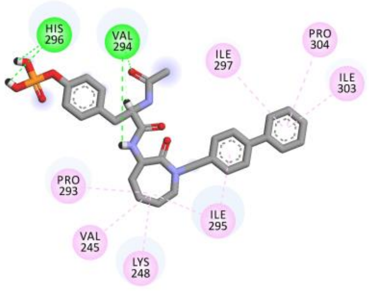

DB04495

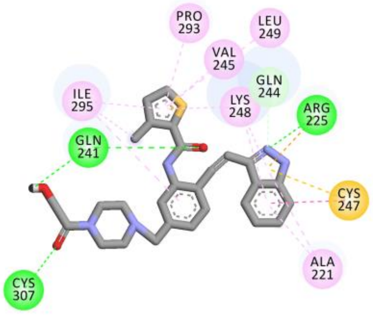

DB16637

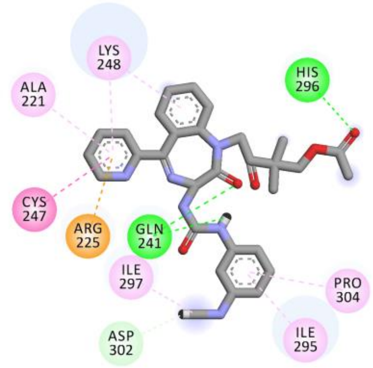

DB17270

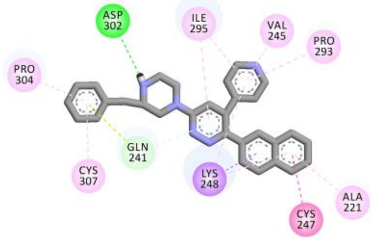

DB01988

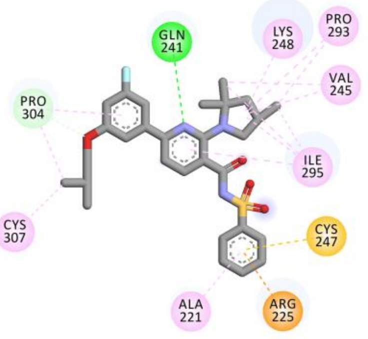

DB16262

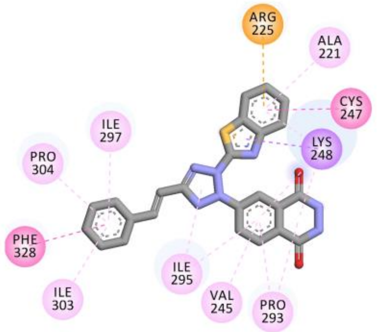

DB01897

Figure S1. *Continued.*

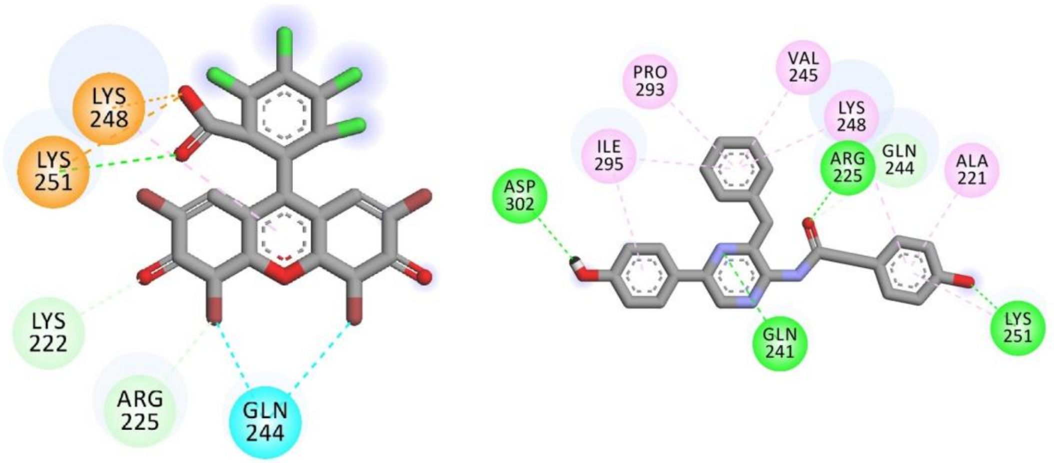

DB13911 **DB04049**

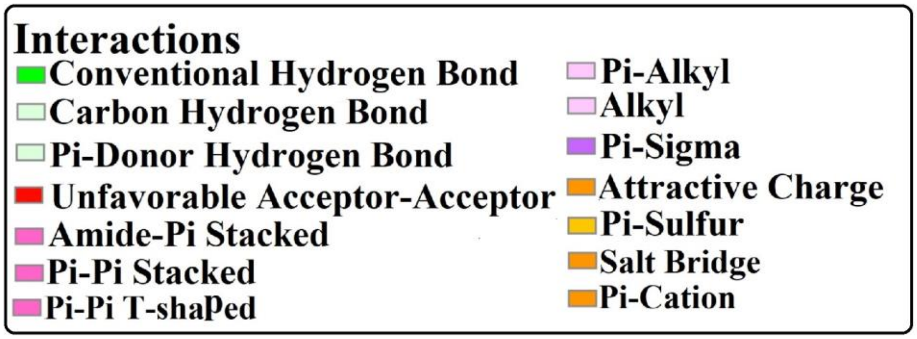

Figure S1. *Continued.*

Table S1. The anticipated quick and moderate docking scores (in kcal/mol) for the top 1301 drug candidates and 1D9 towards VP35 [a].

No.	Compound Code	Docking Score (kcal/mol)		No.	Compound Code	Docking Score (kcal/mol)	
		Quick	Moderate			Quick	Moderate
	**1D9**	**–6.5**	**–6.4**	46	DB15587	–8.6	–8.8
1	DB07424	–9.9	–10.4	47	DB01411	–8.6	–8.8
2	DB14875	−9.7	−10.3	48	DB02033	–8.6	–8.7
3	DB08858	–10.5	–10.0	49	DB01761	–8.6	–8.7
4	DB11183	–9.9	–9.9	50	DB17109	–8.5	–8.7
5	DB09267	−8.6	–9.9	51	DB03076	–8.5	–8.7
6	DB03146	–9.3	–9.8	52	DB15822	–8.5	–8.7
7	DB12051	–8.5	–9.7	53	DB02353	–8.5	–8.7
8	DB06705	–9.8	–9.6	54	DB14950	–8.5	–8.6
9	DB02809	–9.6	–9.4	55	DB02329	–8.5	–8.6
10	DB03948	–9.4	–9.4	56	DB02331	–8.5	–8.6
11	DB06448	–8.4	–9.4	57	DB07460	–8.5	–8.6
12	DB11493	–9.3	–9.4	58	DB16244	–8.5	–8.6
13	DB04285	–8.4	–9.3	59	DB15047	–8.4	–8.6
14	DB09335	–9.2	–9.2	60	DB04032	–8.4	–8.6
15	DB16024	–9.2	–9.2	61	DB04408	–8.4	–8.6
16	DB03104	–8.4	–9.2	62	DB07062	–8.4	–8.5
17	DB04495	–9.0	–9.2	63	DB14883	–8.4	–8.5
18	DB05340	–8.9	–9.2	64	DB03358	–8.4	–8.5
19	DB01988	–9.0	–9.2	65	DB00153	–8.4	–8.5
20	DB01897	–8.9	–9.1	66	DB04859	–8.4	–8.5
21	DB13911	–9.1	–9.1	67	DB17021	–8.4	–8.5
22	DB02322	–7.0	–9.0	68	DB13943	–8.4	–8.5
23	DB04049	–8.9	–9.0	69	DB07875	–8.4	–8.5
24	DB11611	–8.9	–9.0	70	DB11977	–8.4	–8.5
25	DB16637	–8.4	–9.0	71	DB06974	–8.3	–8.4
26	DB08180	–8.8	–9.0	72	DB17059	–8.3	–8.4
27	DB14779	–8.8	–9.0	73	DB12228	–8.3	–8.4
28	DB15310	–8.8	–8.9	74	DB17197	–8.3	–8.4
29	DB02015	–8.2	–8.9	75	DB03433	–8.3	–8.4
30	DB09275	–8.8	–8.9	76	DB12336	–8.3	–8.4
31	DB17270	–8.3	–8.9	77	DB07691	–8.3	–8.4
32	DB15291	–8.8	–8.9	78	DB12424	–8.3	–8.4
33	DB06367	–8.8	–8.9	79	DB16255	–8.3	–8.4
34	DB06595	–8.7	–8.9	80	DB05608	–8.3	–8.4
35	DB15193	–8.7	–8.9	81	DB12649	–8.3	–8.4
36	DB02723	–8.7	–8.9	82	DB15075	–8.3	–8.4
37	DB15416	–8.7	–8.9	83	DB00984	–8.3	–8.4
38	DB12511	–8.1	–8.8	84	DB02137	–8.3	–8.4
39	DB00872	–8.7	–8.8	85	DB02633	–8.3	–8.4
40	DB15385	–8.7	–8.8	86	DB14664	–8.3	–8.4
41	DB00743	–8.7	–8.8	87	DB16844	–8.3	–8.4
42	DB00563	–8.6	–8.8	88	DB14653	–8.3	–8.3
43	DB09138	–8.6	–8.8	89	DB05490	–8.3	–8.3
44	DB15068	–8.6	–8.8	90	DB11651	–8.3	–8.3
45	DB13911	−8.9	−8.8	91	DB14208	–8.3	–8.3

Table S1. *Continued.*

No.	Compound Code	Docking Score (kcal/mol)		No.	Compound Code	Docking Score (kcal/mol)	
		Quick	Moderate			Quick	Moderate
92	DB12491	–8.6	–8.3	139	DB12877	–8.1	–8.1
93	DB12556	–8.3	–8.3	140	DB16038	–8.1	–8.1
94	DB15356	–8.3	–8.3	141	DB07817	–8.0	–8.1
95	DB16758	–8.3	–8.3	142	DB11691	–8.0	–8.1
96	DB04288	–8.2	–8.3	143	DB12121	–8.0	–8.1
97	DB03084	–8.2	–8.3	144	DB12640	–8.0	–8.1
98	DB06883	–8.2	–8.3	145	DB14209	–8.0	–8.1
99	DB08173	–8.2	–8.3	146	DB14632	–8.0	–8.1
100	DB11591	–8.2	–8.3	147	DB15039	–8.0	–8.1
101	DB13640	–8.2	–8.3	148	DB15688	–8.0	–8.1
102	DB05263	–8.2	–8.3	149	DB16184	–8.0	–8.1
103	DB08387	–8.2	–8.3	150	DB04038	–8.0	–8.1
104	DB11800	–8.2	–8.3	151	DB08143	–8.0	–8.1
105	DB16047	–8.2	–8.3	152	DB12388	–8.0	–8.1
106	DB03642	–8.2	–8.3	153	DB15034	–8.0	–8.1
107	DB07700	–8.2	–8.3	154	DB15585	–8.0	–8.1
108	DB11809	–8.2	–8.3	155	DB16875	–8.0	–8.1
109	DB01395	–8.2	–8.2	156	DB00351	–8.0	–8.1
110	DB09233	–8.2	–8.2	157	DB06997	–8.0	–8.1
111	DB12067	–8.2	–8.2	158	DB15775	–8.0	–8.1
112	DB15821	–8.2	–8.2	159	DB01134	–8.0	–8.1
113	DB12522	–8.2	–8.2	160	DB01166	–8.0	–8.1
114	DB16262	–8.1	–8.2	161	DB02852	–8.0	–8.1
115	DB07833	–8.1	–8.2	162	DB12024	–8.0	–8.1
116	DB15273	–8.1	–8.2	163	DB05075	–8.0	–8.1
117	DB00210	–8.1	–8.2	164	DB07607	–7.9	–8.1
118	DB02741	–8.1	–8.2	165	DB12355	–7.9	–8.1
119	DB03231	–8.1	–8.2	166	DB14045	–7.9	–8.1
120	DB05410	–8.1	–8.2	167	DB15614	–7.9	–8.1
121	DB13954	–8.1	–8.2	168	DB00562	–7.9	–8.1
122	DB06251	–8.1	–8.2	169	DB04839	–7.9	–8.1
123	DB08962	–8.1	–8.2	170	DB08386	–7.9	–8.1
124	DB11805	–8.1	–8.2	171	DB11995	–7.9	–8.1
125	DB13947	–8.1	–8.2	172	DB14038	–7.9	–8.1
126	DB16256	–8.1	–8.2	173	DB14125	–7.9	–8.1
127	DB11742	–8.1	–8.2	174	DB14765	–7.9	–8.1
128	DB07145	–8.1	–8.2	175	DB01993	–7.9	–8.0
129	DB07252	–8.1	–8.2	176	DB14976	–7.9	–8.0
130	DB13014	–8.1	–8.2	177	DB12513	–7.9	–8.0
131	DB14659	–8.1	–8.2	178	DB14541	–7.9	–8.0
132	DB02051	–8.1	–8.2	179	DB15903	–7.9	–8.0
133	DB07136	–8.1	–8.2	180	DB16117	–7.9	–8.0
134	DB07872	–8.1	–8.2	181	DB16272	–7.9	–8.0
135	DB06435	–8.1	–8.1	182	DB17117	–7.9	–8.0
136	DB09319	–8.1	–8.1	183	DB01990	–8.1	–8.0
137	DB15594	−9.3	−9.4	184	DB03932	–8.1	–8.0
138	DB04698	–8.1	–8.1	185	DB13664	–7.9	–8.0

Table S1. *Continued.*

No.	Compound Code	Docking Score (kcal/mol)		No.	Compound Code	Docking Score (kcal/mol)	
		Quick	Moderate			Quick	Moderate
186	DB13830	–7.9	–8.0	233	DB06844	–7.8	–7.9
187	DB00471	–7.9	–8.0	234	DB07256	–7.8	–7.8
188	DB02388	–7.9	–8.0	235	DB14790	–7.8	–7.8
189	DB08232	–7.9	–8.0	236	DB16874	–7.8	–7.8
190	DB00169	–7.9	–8.0	237	DB06677	–7.7	–7.8
191	DB02545	–7.9	–8.0	238	DB08305	–7.7	–7.8
192	DB12756	–7.9	–8.0	239	DB11734	–7.7	–7.8
193	DB12764	–7.9	–8.0	240	DB14766	–7.7	–7.8
194	DB08353	–7.9	–8.0	241	DB15883	–7.7	–7.8
195	DB11663	–7.9	–7.9	242	DB00146	–7.7	–7.8
196	DB13090	–7.9	–7.9	243	DB01459	–7.7	–7.8
197	DB15177	–7.9	–7.9	244	DB04759	–7.7	–7.8
198	DB07189	–7.8	–7.9	245	DB07127	–7.7	–7.8
199	DB11698	–7.8	–7.9	246	DB12651	–7.7	–7.8
200	DB15490	–7.8	–7.9	247	DB12729	–7.7	–7.8
201	DB01416	–7.8	–7.9	248	DB12309	–7.7	–7.8
202	DB01813	–7.8	–7.9	249	DB12963	–7.7	–7.8
203	DB03878	–7.8	–7.9	250	DB16058	–7.7	–7.8
204	DB04340	–7.8	–7.9	251	DB17141	–7.7	–7.8
205	DB07605	–7.8	–7.9	252	DB02177	–7.7	–7.8
206	DB16660	–7.8	–7.9	253	DB13036	–7.7	–7.8
207	DB15156	–7.8	–7.9	254	DB06962	–7.7	–7.8
208	DB08875	–7.8	–7.9	255	DB08248	–7.7	–7.8
209	DB13185	–7.8	–7.9	256	DB16253	–7.7	–7.8
210	DB07220	–7.8	–7.9	257	DB08362	–7.7	–7.8
211	DB16239	–7.8	–7.9	258	DB12345	–7.7	–7.8
212	DB06494	–7.8	–7.9	259	DB12743	–7.7	–7.8
213	DB08304	–7.8	–7.9	260	DB13237	–7.7	–7.8
214	DB14850	–7.8	–7.9	261	DB16851	–7.7	–7.8
215	DB16162	–7.8	–7.9	262	DB08221	–7.7	–7.8
216	DB02567	–7.8	–7.9	263	DB15056	–7.7	–7.8
217	DB08242	–7.8	–7.9	264	DB03044	–7.7	–7.8
218	DB14774	–7.8	–7.9	265	DB03571	–7.7	–7.8
219	DB04903	–7.8	–7.9	266	DB08561	–7.7	–7.8
220	DB13931	–7.8	–7.9	267	DB14210	–7.7	–7.8
221	DB16001	–7.8	–7.9	268	DB16095	–7.7	–7.8
222	DB02200	–7.8	–7.9	269	DB00974	–7.7	–7.8
223	DB05611	–7.8	–7.9	270	DB07837	–7.7	–7.8
224	DB15670	–7.8	–7.9	271	DB11877	–7.7	–7.8
225	DB16843	–7.8	–7.9	272	DB14679	–7.7	–7.8
226	DB01772	–7.8	–7.9	273	DB01888	–7.7	–7.8
227	DB07253	–7.8	–7.9	274	DB03591	–7.7	–7.7
228	DB07261	–7.8	–7.9	275	DB04765	–7.7	–7.7
229	DB15035	–7.8	–7.9	276	DB04888	–7.7	–7.7
230	DB07688	–7.8	–7.9	277	DB07853	–7.7	–7.7
231	DB04575	–7.8	–7.9	278	DB08096	–7.7	–7.7
232	DB14895	–7.8	–7.9	279	DB08125	–7.7	–7.7

Table S1. *Continued.*

No.	Compound Code	Docking Score (kcal/mol)		No.	Compound Code	Docking Score (kcal/mol)	
		Quick	Moderate			Quick	Moderate
280	DB08213	–7.7	–7.7	327	DB15292	–7.6	–7.7
281	DB11814	–7.7	–7.7	328	DB01434	–7.6	–7.7
282	DB15287	–7.7	–7.7	329	DB13981	–7.6	–7.7
283	DB03837	–7.7	–7.7	330	DB03944	–7.6	–7.6
284	DB04696	–7.7	–7.7	331	DB05983	–7.6	–7.6
285	DB16214	–7.7	–7.7	332	DB08707	–7.6	–7.6
286	DB07270	–7.7	–7.7	333	DB16035	–7.6	–7.6
287	DB07697	–7.7	–7.7	334	DB01640	–7.6	–7.6
288	DB13797	–7.6	–7.7	335	DB06075	–7.6	–7.6
289	DB02848	–7.6	–7.7	336	DB08358	–7.6	–7.6
290	DB05966	–7.6	–7.7	337	DB13097	–7.6	–7.6
291	DB07685	–7.6	–7.7	338	DB00276	–7.6	–7.6
292	DB11729	–7.6	–7.7	339	DB07717	–7.6	–7.6
293	DB12381	–7.6	–7.7	340	DB08031	–7.6	–7.6
294	DB01138	–7.6	–7.7	341	DB12776	–7.6	–7.6
295	DB01254	–7.6	–7.7	342	DB13953	–7.6	–7.6
296	DB07588	–7.6	–7.7	343	DB16344	–7.6	–7.6
297	DB11511	–7.6	–7.7	344	DB17235	–7.6	–7.6
298	DB12012	–7.6	–7.7	345	DB00876	–7.6	–7.6
299	DB14845	–7.6	–7.7	346	DB07049	–7.6	–7.6
300	DB00496	–7.6	–7.7	347	DB12888	–7.6	–7.6
301	DB00906	–7.6	–7.7	348	DB13215	–7.6	–7.6
302	DB01216	–7.6	–7.7	349	DB13685	–7.6	–7.6
303	DB03234	–7.6	–7.7	350	DB05686	–7.6	–7.6
304	DB04705	–7.6	–7.7	351	DB08354	–7.6	–7.6
305	DB06155	–7.6	–7.7	352	DB11808	–7.6	–7.6
306	DB08416	–7.6	–7.7	353	DB13042	–7.6	–7.6
307	DB12612	–7.6	–7.7	354	DB16054	–7.6	–7.6
308	DB12812	–7.6	–7.7	355	DB16124	–7.6	–7.6
309	DB14070	–7.6	–7.7	356	DB17159	–7.6	–7.6
310	DB14543	–7.6	–7.7	357	DB00222	–7.6	–7.6
311	DB15396	–7.6	–7.7	358	DB01016	–7.6	–7.6
312	DB16216	–7.6	–7.7	359	DB02479	–7.6	–7.6
313	DB00843	–7.6	–7.7	360	DB03748	–7.6	–7.6
314	DB04693	–7.6	–7.7	361	DB05185	–7.6	–7.6
315	DB12271	–7.6	–7.7	362	DB06684	–7.6	–7.6
316	DB14639	–7.6	–7.7	363	DB11978	–7.6	–7.6
317	DB15282	–7.6	–7.7	364	DB17131	–7.6	–7.6
318	DB06789	–7.6	–7.7	365	DB01200	–7.6	–7.6
319	DB11348	–7.6	–7.7	366	DB01251	–7.5	–7.6
320	DB15630	–7.6	–7.7	367	DB01443	–7.5	–7.6
321	DB00820	–7.6	–7.7	368	DB07453	–7.5	–7.6
322	DB01349	–7.6	–7.7	369	DB07724	–7.5	–7.6
323	DB08804	–7.6	–7.7	370	DB09268	–7.5	–7.6
324	DB09030	–7.6	–7.7	371	DB11818	–7.5	–7.6
325	DB11622	–7.6	–7.7	372	DB12221	–7.5	–7.6
326	DB12887	–7.6	–7.7	373	DB16043	–7.5	–7.6

Table S1. *Continued.*

No.	Compound Code	Docking Score (kcal/mol)		No.	Compound Code	Docking Score (kcal/mol)	
		Quick	Moderate			Quick	Moderate
374	DB06117	–7.5	–7.6	421	DB16074	–7.5	–7.5
375	DB07192	–7.5	–7.6	422	DB16930	–7.5	–7.5
376	DB08547	–7.5	–7.6	423	DB01810	–7.5	–7.5
377	DB15245	–7.5	–7.6	424	DB04739	–7.5	–7.5
378	DB16108	–7.5	–7.6	425	DB11526	–7.5	–7.5
379	DB02118	–7.5	–7.6	426	DB12574	–7.5	–7.5
380	DB12196	–7.5	–7.6	427	DB13487	–7.5	–7.5
381	DB14982	–7.5	–7.6	428	DB14948	–7.5	–7.5
382	DB01342	–7.5	–7.6	429	DB16068	–7.5	–7.5
383	DB03141	–7.5	–7.6	430	DB16185	–7.5	–7.5
384	DB06212	–7.5	–7.6	431	DB16237	–7.5	–7.5
385	DB08220	–7.5	–7.6	432	DB16739	–7.5	–7.5
386	DB12234	–7.5	–7.6	433	DB00320	–7.5	–7.5
387	DB14676	–7.5	–7.6	434	DB07247	–7.5	–7.5
388	DB15297	–7.5	–7.6	435	DB07547	–7.5	–7.5
389	DB15393	–7.5	–7.6	436	DB08303	–7.5	–7.5
390	DB04154	–7.5	–7.5	437	DB16761	–7.5	–7.5
391	DB04540	–7.5	–7.5	438	DB17136	–7.5	–7.5
392	DB06442	–7.5	–7.5	439	DB00619	–7.5	–7.5
393	DB07029	–7.5	–7.5	440	DB03903	–7.5	–7.5
394	DB08556	–7.5	–7.5	441	DB08867	–7.5	–7.5
395	DB12194	–7.5	–7.5	442	DB04764	–7.5	–7.5
396	DB12415	–7.5	–7.5	443	DB08543	–7.4	–7.5
397	DB12914	–7.5	–7.5	444	DB12694	–7.4	–7.5
398	DB14993	–7.5	–7.5	445	DB16891	–7.4	–7.5
399	DB04330	–7.5	–7.5	446	DB03712	–7.4	–7.5
400	DB05450	–7.5	–7.5	447	DB04706	–7.4	–7.5
401	DB11773	–7.5	–7.5	448	DB05804	–7.4	–7.5
402	DB13003	–7.5	–7.5	449	DB07626	–7.4	–7.5
403	DB15055	–7.5	–7.5	450	DB12129	–7.4	–7.5
404	DB00355	–7.5	–7.5	451	DB12644	–7.4	–7.5
405	DB00603	–7.5	–7.5	452	DB16906	–7.4	–7.5
406	DB01126	–7.5	–7.5	453	DB02702	–7.4	–7.5
407	DB07414	–7.5	–7.5	454	DB06626	–7.4	–7.5
408	DB08278	–7.5	–7.5	455	DB08111	–7.4	–7.5
409	DB09164	–7.5	–7.5	456	DB12204	–7.4	–7.5
410	DB12696	–7.5	–7.5	457	DB13454	–7.4	–7.5
411	DB16883	–7.5	–7.5	458	DB01410	–7.4	–7.4
412	DB02008	–7.5	–7.5	459	DB06414	–7.4	–7.4
413	DB02258	–7.5	–7.5	460	DB07090	–7.4	–7.4
414	DB02877	–7.5	–7.5	461	DB07194	–7.4	–7.4
415	DB08299	–7.5	–7.5	462	DB08549	–7.4	–7.4
416	DB08444	–7.5	–7.5	463	DB11984	–7.4	–7.4
417	DB11878	–7.5	–7.5	464	DB12903	–7.4	–7.4
418	DB12514	–7.5	–7.5	465	DB13089	–7.4	–7.4
419	DB12999	–7.5	–7.5	466	DB16707	–7.4	–7.4
420	DB14929	–7.5	–7.5	467	DB03233	–7.4	–7.4

Table S1. *Continued.*

No.	Compound Code	Docking Score (kcal/mol)		No.	Compound Code	Docking Score (kcal/mol)	
		Quick	Moderate			Quick	Moderate
468	DB04186	–7.4	–7.4	515	DB06597	–7.4	–7.4
469	DB06140	–7.4	–7.4	516	DB07183	–7.4	–7.4
470	DB06486	–7.4	–7.4	517	DB07471	–7.4	–7.4
471	DB06972	–7.4	–7.4	518	DB08097	–7.4	–7.4
472	DB07843	–7.4	–7.4	519	DB08705	–7.4	–7.4
473	DB08560	–7.4	–7.4	520	DB11859	–7.4	–7.4
474	DB09477	–7.4	–7.4	521	DB12703	–7.4	–7.4
475	DB11904	–7.4	–7.4	522	DB13059	–7.4	–7.4
476	DB14902	–7.4	–7.4	523	DB13591	–7.4	–7.4
477	DB16226	–7.4	–7.4	524	DB14918	–7.4	–7.4
478	DB08122	–7.4	–7.4	525	DB16217	–7.3	–7.4
479	DB11987	–7.4	–7.4	526	DB04037	–7.3	–7.3
480	DB14069	–7.4	–7.4	527	DB04704	–7.3	–7.3
481	DB14878	–7.4	–7.4	528	DB07128	–7.3	–7.3
482	DB15448	–7.4	–7.4	529	DB12149	–7.3	–7.3
483	DB01347	–7.4	–7.4	530	DB14678	–7.3	–7.3
484	DB07430	–7.4	–7.4	531	DB15011	–7.3	–7.3
485	DB12112	–7.4	–7.4	532	DB15639	–7.3	–7.3
486	DB04405	–7.4	–7.4	533	DB15647	–7.3	–7.3
487	DB13040	–7.4	–7.4	534	DB17049	–7.3	–7.3
488	DB15121	–7.4	–7.4	535	DB05713	–7.3	–7.3
489	DB16252	–7.4	–7.4	536	DB06276	–7.3	–7.3
490	DB00414	–7.4	–7.4	537	DB06347	–7.3	–7.3
491	DB02799	–7.4	–7.4	538	DB11922	–7.3	–7.3
492	DB13094	–7.4	–7.4	539	DB14673	–7.3	–7.3
493	DB14662	–7.4	–7.4	540	DB03577	–7.3	–7.3
494	DB15254	–7.4	–7.4	541	DB04788	–7.3	–7.3
495	DB16321	–7.4	–7.4	542	DB09298	–7.3	–7.3
496	DB04741	–7.4	–7.4	543	DB12670	–7.3	–7.3
497	DB08384	–7.4	–7.4	544	DB14538	–7.3	–7.3
498	DB11863	–7.4	–7.4	545	DB16232	–7.3	–7.3
499	DB12910	–7.4	–7.4	546	DB17269	–7.3	–7.3
500	DB15124	–7.4	–7.4	547	DB00421	–7.3	–7.3
501	DB00737	–7.4	–7.4	548	DB02300	–7.3	–7.3
502	DB01879	–7.4	–7.4	549	DB02827	–7.3	–7.3
503	DB04724	–7.4	–7.4	550	DB03453	–7.3	–7.3
504	DB07076	–7.4	–7.4	551	DB04289	–7.3	–7.3
505	DB13791	–7.4	–7.4	552	DB12168	–7.3	–7.3
506	DB03549	–7.4	–7.4	553	DB12512	–7.3	–7.3
507	DB03696	–7.4	–7.4	554	DB15640	–7.3	–7.3
508	DB07142	–7.4	–7.4	555	DB00253	–7.3	–7.3
509	DB07827	–7.4	–7.4	556	DB01946	–7.3	–7.3
510	DB12387	–7.4	–7.4	557	DB06876	–7.3	–7.3
511	DB01357	–7.4	–7.4	558	DB07175	–7.3	–7.3
512	DB04392	–7.4	–7.4	559	DB13019	–7.3	–7.3
513	DB04727	–7.4	–7.4	560	DB15308	–7.3	–7.3
514	DB06163	–7.4	–7.4	561	DB16846	–7.3	–7.3

Table S1. *Continued.*

No.	Compound Code	Docking Score (kcal/mol)		No.	Compound Code	Docking Score (kcal/mol)	
		Quick	Moderate			Quick	Moderate
562	DB04186	–7.3	–7.3	609	DB06597	–7.3	–7.2
563	DB06140	–7.3	–7.3	610	DB07183	–7.3	–7.2
564	DB06486	–7.3	–7.3	611	DB07471	–7.3	–7.2
565	DB06972	–7.3	–7.3	612	DB08097	–7.3	–7.2
566	DB07843	–7.3	–7.3	613	DB08705	–7.3	–7.2
567	DB08560	–7.3	–7.3	614	DB11859	–7.3	–7.2
568	DB09477	–7.3	–7.3	615	DB12703	–7.3	–7.2
569	DB11904	–7.3	–7.3	616	DB13059	–7.3	–7.2
570	DB14902	–7.3	–7.3	617	DB13591	–7.3	–7.2
571	DB16226	–7.3	–7.3	618	DB14918	–7.3	–7.2
572	DB08122	–7.3	–7.3	619	DB16217	–7.3	–7.2
573	DB11987	–7.3	–7.3	620	DB04037	–7.3	–7.2
574	DB14069	–7.3	–7.3	621	DB04704	–7.3	–7.2
575	DB14878	–7.3	–7.3	622	DB07128	–7.3	–7.2
576	DB15448	–7.3	–7.3	623	DB12149	–7.3	–7.2
577	DB01347	–7.3	–7.3	624	DB14678	–7.3	–7.2
578	DB07430	–7.3	–7.3	625	DB15011	–7.3	–7.2
579	DB12112	–7.3	–7.3	626	DB15639	–7.3	–7.2
580	DB04405	–7.3	–7.3	627	DB15647	–7.2	–7.2
581	DB13040	–7.3	–7.3	628	DB17049	–7.2	–7.2
582	DB15121	–7.3	–7.3	629	DB05713	–7.2	–7.2
583	DB16252	–7.3	–7.3	630	DB06276	–7.2	–7.2
584	DB00414	–7.3	–7.3	631	DB06347	–7.2	–7.2
585	DB02799	–7.3	–7.3	632	DB11922	–7.2	–7.2
586	DB13094	–7.3	–7.3	633	DB14673	–7.2	–7.2
587	DB14662	–7.3	–7.3	634	DB03577	–7.2	–7.2
588	DB15254	–7.3	–7.2	635	DB04788	–7.2	–7.2
589	DB16321	–7.3	–7.2	636	DB09298	–7.2	–7.2
590	DB04741	–7.3	–7.2	637	DB12670	–7.2	–7.2
591	DB08384	–7.3	–7.2	638	DB14538	–7.2	–7.2
592	DB11863	–7.3	–7.2	639	DB16232	–7.2	–7.2
593	DB12910	–7.3	–7.2	640	DB17269	–7.2	–7.2
594	DB15124	–7.3	–7.2	641	DB00421	–7.2	–7.2
595	DB00737	–7.3	–7.2	642	DB02300	–7.2	–7.2
596	DB01879	–7.3	–7.2	643	DB02827	–7.2	–7.2
597	DB04724	–7.3	–7.2	644	DB03453	–7.2	–7.2
598	DB07076	–7.3	–7.2	645	DB04289	–7.2	–7.2
599	DB13791	–7.3	–7.2	646	DB12168	–7.2	–7.2
600	DB03549	–7.3	–7.2	647	DB12512	–7.2	–7.2
601	DB03696	–7.3	–7.2	648	DB15640	–7.2	–7.2
602	DB07142	–7.3	–7.2	649	DB00253	–7.2	–7.2
603	DB07827	–7.3	–7.2	650	DB01946	–7.2	–7.2
604	DB12387	–7.3	–7.2	651	DB06876	–7.2	–7.2
605	DB01357	–7.3	–7.2	652	DB07175	–7.2	–7.2
606	DB04392	–7.3	–7.2	653	DB13019	–7.2	–7.2
607	DB04727	–7.3	–7.2	654	DB15308	–7.2	–7.2
608	DB06163	–7.3	–7.2	655	DB16846	–7.2	–7.2

Table S1. *Continued.*

No.	Compound Code	Docking Score (kcal/mol)		No.	Compound Code	Docking Score (kcal/mol)	
		Quick	Moderate			Quick	Moderate
656	DB16347	–7.2	–7.2	703	DB11636	–7.2	–7.1
657	DB16650	–7.2	–7.2	704	DB14715	–7.2	–7.1
658	DB17015	–7.2	–7.2	705	DB14961	–7.2	–7.1
659	DB04258	–7.2	–7.2	706	DB14995	–7.2	–7.1
660	DB06080	–7.2	–7.2	707	DB15984	–7.2	–7.1
661	DB06137	–7.2	–7.2	708	DB16346	–7.2	–7.1
662	DB06780	–7.2	–7.2	709	DB00197	–7.2	–7.1
663	DB07382	–7.2	–7.2	710	DB02850	–7.2	–7.1
664	DB07811	–7.2	–7.2	711	DB07222	–7.2	–7.1
665	DB12693	–7.2	–7.2	712	DB07557	–7.2	–7.1
666	DB13035	–7.2	–7.2	713	DB08005	–7.2	–7.1
667	DB15048	–7.2	–7.2	714	DB08400	–7.2	–7.1
668	DB01339	–7.2	–7.2	715	DB11393	–7.1	–7.1
669	DB01948	–7.2	–7.2	716	DB11703	–7.1	–7.1
670	DB07943	–7.2	–7.2	717	DB11775	–7.1	–7.1
671	DB08349	–7.2	–7.2	718	DB12361	–7.1	–7.1
672	DB08553	–7.2	–7.2	719	DB12382	–7.1	–7.1
673	DB12225	–7.2	–7.2	720	DB13993	–7.1	–7.1
674	DB13602	–7.2	–7.2	721	DB15009	–7.1	–7.1
675	DB02360	–7.2	–7.1	722	DB16141	–7.1	–7.1
676	DB05403	–7.2	–7.1	723	DB16254	–7.1	–7.1
677	DB05467	–7.2	–7.1	724	DB02473	–7.1	–7.1
678	DB06933	–7.2	–7.1	725	DB02616	–7.1	–7.1
679	DB07778	–7.2	–7.1	726	DB03964	–7.1	–7.1
680	DB07804	–7.2	–7.1	727	DB06963	–7.1	–7.1
681	DB11529	–7.2	–7.1	728	DB07514	–7.1	–7.1
682	DB11721	–7.2	–7.1	729	DB07629	–7.1	–7.1
683	DB12193	–7.2	–7.1	730	DB08535	–7.1	–7.1
684	DB12211	–7.2	–7.1	731	DB08743	–7.1	–7.1
685	DB13675	–7.2	–7.1	732	DB09034	–7.1	–7.1
686	DB16098	–7.2	–7.1	733	DB11385	–7.1	–7.1
687	DB04869	–7.2	–7.1	734	DB12200	–7.1	–7.1
688	DB07586	–7.2	–7.1	735	DB12622	–7.1	–7.1
689	DB07973	–7.2	–7.1	736	DB13101	–7.1	–7.1
690	DB08126	–7.2	–7.1	737	DB14626	–7.1	–7.1
691	DB08233	–7.2	–7.1	738	DB16241	–7.1	–7.1
692	DB09137	–7.2	–7.1	739	DB00957	–7.1	–7.1
693	DB11778	–7.2	–7.1	740	DB07148	–7.1	–7.1
694	DB17384	–7.2	–7.1	741	DB08052	–7.1	–7.1
695	DB06708	–7.2	–7.1	742	DB12001	–7.1	–7.1
696	DB13223	–7.2	–7.1	743	DB12590	–7.1	–7.1
697	DB13520	–7.2	–7.1	744	DB14624	–7.1	–7.1
698	DB16834	–7.2	–7.1	745	DB14773	–7.1	–7.1
699	DB17096	–7.2	–7.1	746	DB16944	–7.1	–7.1
700	DB00568	–7.2	–7.1	747	DB00451	–7.1	–7.1
701	DB00838	–7.2	–7.1	748	DB00773	–7.1	–7.1
702	DB06401	–7.2	–7.1	749	DB01501	–7.1	–7.1

Table S1. *Continued.*

No.	Compound Code	Docking Score (kcal/mol)		No.	Compound Code	Docking Score (kcal/mol)	
		Quick	Moderate			Quick	Moderate
750	DB04477	–7.1	–7.1	797	DB07945	–7.1	–7.0
751	DB06581	–7.1	–7.1	798	DB13061	–7.1	–7.0
752	DB06660	–7.1	–7.1	799	DB14675	–7.1	–7.0
753	DB00481	–7.1	–7.1	800	DB16267	–7.1	–7.0
754	DB02253	–7.1	–7.1	801	DB16304	–7.1	–7.0
755	DB03670	–7.1	–7.1	802	DB16343	–7.1	–7.0
756	DB04213	–7.1	–7.1	803	DB16879	–7.1	–7.0
757	DB05015	–7.1	–7.1	804	DB07839	–7.1	–7.0
758	DB06249	–7.1	–7.1	805	DB08095	–7.1	–7.0
759	DB08457	–7.1	–7.1	806	DB08385	–7.1	–7.0
760	DB08971	–7.1	–7.1	807	DB09382	–7.1	–7.0
761	DB16044	–7.1	–7.1	808	DB11913	–7.1	–7.0
762	DB00394	–7.1	–7.1	809	DB12135	–7.1	–7.0
763	DB00739	–7.1	–7.1	810	DB13158	–7.1	–7.0
764	DB00896	–7.1	–7.1	811	DB13857	–7.1	–7.0
765	DB04241	–7.1	–7.1	812	DB14916	–7.1	–7.0
766	DB04430	–7.1	–7.1	813	DB15036	–7.1	–7.0
767	DB07312	–7.1	–7.1	814	DB00310	–7.1	–7.0
768	DB07403	–7.1	–7.1	815	DB01941	–7.1	–7.0
769	DB07792	–7.1	–7.1	816	DB02854	–7.1	–7.0
770	DB12048	–7.1	–7.1	817	DB07031	–7.1	–7.0
771	DB12457	–7.1	–7.1	818	DB15861	–7.1	–7.0
772	DB14637	–7.1	–7.1	819	DB03480	–7.1	–7.0
773	DB14943	–7.1	–7.1	820	DB07015	–7.1	–7.0
774	DB15959	–7.1	–7.1	821	DB07075	–7.1	–7.0
775	DB16137	–7.1	–7.1	822	DB08042	–7.1	–7.0
776	DB03082	–7.1	–7.0	823	DB08340	–7.1	–7.0
777	DB06693	–7.1	–7.0	824	DB08555	–7.1	–7.0
778	DB07765	–7.1	–7.0	825	DB12158	–7.1	–7.0
779	DB12165	–7.1	–7.0	826	DB14801	–7.1	–7.0
780	DB15401	–7.1	–7.0	827	DB15362	–7.1	–7.0
781	DB05767	–7.1	–7.0	828	DB16308	–7.1	–7.0
782	DB06940	–7.1	–7.0	829	DB16330	–7.1	–7.0
783	DB07020	–7.1	–7.0	830	DB02222	–7.1	–7.0
784	DB07698	–7.1	–7.0	831	DB03598	–7.1	–7.0
785	DB07861	–7.1	–7.0	832	DB04142	–7.1	–7.0
786	DB09074	–7.1	–7.0	833	DB05532	–7.1	–7.0
787	DB11796	–7.1	–7.0	834	DB06908	–7.1	–7.0
788	DB12272	–7.1	–7.0	835	DB07269	–7.1	–7.0
789	DB13528	–7.1	–7.0	836	DB07570	–7.1	–7.0
790	DB14844	–7.1	–7.0	837	DB07846	–7.1	–7.0
791	DB14986	–7.1	–7.0	838	DB08154	–7.1	–7.0
792	DB15579	–7.1	–7.0	839	DB14657	–7.1	–7.0
793	DB01889	–7.1	–7.0	840	DB16009	–7.1	–7.0
794	DB03262	–7.1	–7.0	841	DB02519	–7.1	–7.0
795	DB06721	–7.1	–7.0	842	DB05812	–7.1	–7.0
796	DB07286	–7.1	–7.0	843	DB05905	–7.1	–7.0

Table S1. *Continued.*

No.	Compound Code	Docking Score (kcal/mol)		No.	Compound Code	Docking Score (kcal/mol)	
		Quick	Moderate			Quick	Moderate
844	DB06134	–7.1	–7.0	891	DB16173	–7.0	–6.9
845	DB06983	–7.1	–7.0	892	DB01406	–7.0	–6.9
846	DB07187	–7.0	–7.0	893	DB01720	–7.0	–6.9
847	DB08731	–7.0	–7.0	894	DB02132	–7.0	–6.9
848	DB11844	–7.0	–7.0	895	DB03331	–7.0	–6.9
849	DB12679	–7.0	–7.0	896	DB03777	–7.0	–6.9
850	DB14823	–7.0	–7.0	897	DB04879	–7.0	–6.9
851	DB15673	–7.0	–7.0	898	DB04960	–7.0	–6.9
852	DB00444	–7.0	–7.0	899	DB06834	–7.0	–6.9
853	DB04177	–7.0	–7.0	900	DB07653	–7.0	–6.9
854	DB04930	–7.0	–7.0	901	DB08057	–7.0	–6.9
855	DB05171	–7.0	–7.0	902	DB08536	–7.0	–6.9
856	DB07275	–7.0	–7.0	903	DB08590	–7.0	–6.9
857	DB07856	–7.0	–7.0	904	DB09183	–7.0	–6.9
858	DB08701	–7.0	–7.0	905	DB14729	–7.0	–6.9
859	DB14654	–7.0	–7.0	906	DB16838	–7.0	–6.9
860	DB16227	–7.0	–7.0	907	DB04852	–7.0	–6.9
861	DB00378	–7.0	–7.0	908	DB06409	–7.0	–6.9
862	DB03523	–7.0	–7.0	909	DB08014	–7.0	–6.9
863	DB07061	–7.0	–7.0	910	DB08631	–7.0	–6.9
864	DB07267	–7.0	–7.0	911	DB08742	–7.0	–6.9
865	DB07680	–7.0	–7.0	912	DB11452	–7.0	–6.9
866	DB11817	–7.0	–7.0	913	DB13129	–7.0	–6.9
867	DB12454	–7.0	–7.0	914	DB00396	–7.0	–6.9
868	DB13600	–7.0	–7.0	915	DB00823	–7.0	–6.9
869	DB13828	–7.0	–7.0	916	DB07216	–7.0	–6.9
870	DB07278	–7.0	–6.9	917	DB07513	–7.0	–6.9
871	DB09296	–7.0	–6.9	918	DB12318	–7.0	–6.9
872	DB11772	–7.0	–6.9	919	DB12562	–7.0	–6.9
873	DB12316	–7.0	–6.9	920	DB14512	–7.0	–6.9
874	DB12322	–7.0	–6.9	921	DB14655	–7.0	–6.9
875	DB16051	–7.0	–6.9	922	DB14989	–7.0	–6.9
876	DB16107	–7.0	–6.9	923	DB15085	–7.0	–6.9
877	DB16266	–7.0	–6.9	924	DB15442	–7.0	–6.9
878	DB16762	–7.0	–6.9	925	DB16774	–7.0	–6.9
879	DB01456	–7.0	–6.9	926	DB17029	–7.0	–6.9
880	DB01586	–7.0	–6.9	927	DB01117	–7.0	–6.9
881	DB02833	–7.0	–6.9	928	DB05990	–7.0	–6.9
882	DB03742	–7.0	–6.9	929	DB07531	–7.0	–6.9
883	DB06497	–7.0	–6.9	930	DB08820	–7.0	–6.9
884	DB07325	–7.0	–6.9	931	DB12784	–7.0	–6.9
885	DB07358	–7.0	–6.9	932	DB15568	–7.0	–6.9
886	DB07373	–7.0	–6.9	933	DB16898	–7.0	–6.9
887	DB12222	–7.0	–6.9	934	DB02038	–7.0	–6.9
888	DB13217	–7.0	–6.9	935	DB03515	–7.0	–6.9
889	DB13276	–7.0	–6.9	936	DB05038	–7.0	–6.9
890	DB14671	–7.0	–6.9	937	DB07162	–7.0	–6.9

Table S1. *Continued.*

No.	Compound Code	Docking Score (kcal/mol)		No.	Compound Code	Docking Score (kcal/mol)	
		Quick	Moderate			Quick	Moderate
938	DB07343	–7.0	–6.9	985	DB07869	–7.0	–6.8
939	DB08246	–7.0	–6.9	986	DB08373	–7.0	–6.8
940	DB11619	–7.0	–6.9	987	DB08656	–7.0	–6.8
941	DB12960	–7.0	–6.9	988	DB12229	–7.0	–6.8
942	DB14035	–7.0	–6.9	989	DB14627	–7.0	–6.8
943	DB16066	–7.0	–6.9	990	DB17099	–7.0	–6.8
944	DB16119	–7.0	–6.9	991	DB00547	–7.0	–6.8
945	DB04089	–7.0	–6.9	992	DB00894	–7.0	–6.8
946	DB06429	–7.0	–6.9	993	DB03957	–7.0	–6.8
947	DB07729	–7.0	–6.9	994	DB08365	–7.0	–6.8
948	DB07835	–7.0	–6.9	995	DB11881	–7.0	–6.8
949	DB11369	–7.0	–6.9	996	DB13682	–7.0	–6.8
950	DB12325	–7.0	–6.9	997	DB14634	–7.0	–6.8
951	DB12720	–7.0	–6.9	998	DB15256	–7.0	–6.8
952	DB13371	–7.0	–6.9	999	DB17163	–6.9	–6.8
953	DB14544	–7.0	–6.9	1000	DB00596	–6.9	–6.8
954	DB14581	–7.0	–6.9	1001	DB04885	–6.9	–6.8
955	DB14810	–7.0	–6.9	1002	DB06362	–6.9	–6.8
956	DB15338	–7.0	–6.9	1003	DB08532	–6.9	–6.8
957	DB02241	–7.0	–6.9	1004	DB08883	–6.9	–6.8
958	DB02973	–7.0	–6.9	1005	DB12611	–6.9	–6.8
959	DB08881	–7.0	–6.9	1006	DB13169	–6.9	–6.8
960	DB11692	–7.0	–6.9	1007	DB16133	–6.9	–6.8
961	DB12719	–7.0	–6.9	1008	DB00635	–6.9	–6.8
962	DB12792	–7.0	–6.9	1009	DB00834	–6.9	–6.8
963	DB16299	–7.0	–6.9	1010	DB01215	–6.9	–6.8
964	DB16331	–7.0	–6.9	1011	DB01252	–6.9	–6.8
965	DB00519	–7.0	–6.9	1012	DB04434	–6.9	–6.8
966	DB00685	–7.0	–6.9	1013	DB08787	–6.9	–6.8
967	DB04044	–7.0	–6.9	1014	DB12550	–6.9	–6.8
968	DB05772	–7.0	–6.9	1015	DB14785	–6.9	–6.8
969	DB05838	–7.0	–6.9	1016	DB15346	–6.9	–6.8
970	DB06160	–7.0	–6.9	1017	DB03583	–6.9	–6.8
971	DB08204	–7.0	–6.9	1018	DB04081	–6.9	–6.8
972	DB08534	–7.0	–6.9	1019	DB07010	–6.9	–6.8
973	DB17055	–7.0	–6.9	1020	DB08039	–6.9	–6.8
974	DB04841	–7.0	–6.9	1021	DB11789	–6.9	–6.8
975	DB05107	–7.0	–6.8	1022	DB11948	–6.9	–6.8
976	DB07117	–7.0	–6.8	1023	DB12180	–6.9	–6.8
977	DB08703	–7.0	–6.8	1024	DB14584	–6.9	–6.8
978	DB12280	–7.0	–6.8	1025	DB14822	–6.9	–6.8
979	DB13277	–7.0	–6.8	1026	DB14949	–6.9	–6.8
980	DB15637	–7.0	–6.8	1027	DB15950	–6.9	–6.8
981	DB00605	–7.0	–6.8	1028	DB04761	–6.9	–6.8
982	DB00814	–7.0	–6.8	1029	DB05520	–6.9	–6.8
983	DB01451	–7.0	–6.8	1030	DB06852	–6.9	–6.8
984	DB07537	–7.0	–6.8	1031	DB07869	–6.9	–6.8

Table S1. *Continued.*

No.	Compound Code	Docking Score (kcal/mol)		No.	Compound Code	Docking Score (kcal/mol)	
		Quick	Moderate			Quick	Moderate
1032	DB08360	–6.9	–6.8	1079	DB11900	–6.9	–6.7
1033	DB08940	–6.9	–6.8	1080	DB12659	–6.9	–6.7
1034	DB12015	–6.9	–6.8	1081	DB15149	–6.9	–6.7
1035	DB15209	–6.9	–6.8	1082	DB00621	–6.9	–6.7
1036	DB17115	–6.9	–6.8	1083	DB04256	–6.9	–6.7
1037	DB01561	–6.9	–6.8	1084	DB05327	–6.9	–6.7
1038	DB03542	–6.9	–6.8	1085	DB06555	–6.9	–6.7
1039	DB12817	–6.9	–6.8	1086	DB06578	–6.9	–6.7
1040	DB14660	–6.9	–6.8	1087	DB07705	–6.9	–6.7
1041	DB16045	–6.9	–6.8	1088	DB11891	–6.9	–6.7
1042	DB16234	–6.9	–6.8	1089	DB12886	–6.9	–6.7
1043	DB01545	–6.9	–6.8	1090	DB13563	–6.9	–6.7
1044	DB02917	–6.9	–6.8	1091	DB14649	–6.9	–6.7
1045	DB04872	–6.9	–6.8	1092	DB15566	–6.9	–6.7
1046	DB07560	–6.9	–6.8	1093	DB04371	–6.9	–6.7
1047	DB07793	–6.9	–6.8	1094	DB05423	–6.9	–6.7
1048	DB08025	–6.9	–6.8	1095	DB07078	–6.9	–6.7
1049	DB12658	–6.9	–6.8	1096	DB07150	–6.9	–6.7
1050	DB13109	–6.9	–6.8	1097	DB07401	–6.9	–6.7
1051	DB13752	–6.9	–6.8	1098	DB07595	–6.9	–6.7
1052	DB16123	–6.9	–6.8	1099	DB08485	–6.9	–6.7
1053	DB03921	–6.9	–6.8	1100	DB08905	–6.9	–6.7
1054	DB03996	–6.9	–6.8	1101	DB12721	–6.9	–6.7
1055	DB05678	–6.9	–6.8	1102	DB12731	–6.9	–6.7
1056	DB05956	–6.9	–6.8	1103	DB12921	–6.9	–6.7
1057	DB06944	–6.9	–6.8	1104	DB14814	–6.9	–6.7
1058	DB07033	–6.9	–6.8	1105	DB15006	–6.9	–6.7
1059	DB07971	–6.9	–6.8	1106	DB16191	–6.9	–6.7
1060	DB11791	–6.9	–6.8	1107	DB00699	–6.9	–6.7
1061	DB12301	–6.9	–6.8	1108	DB01689	–6.9	–6.7
1062	DB12416	–6.9	–6.8	1109	DB02706	–6.9	–6.7
1063	DB13432	–6.9	–6.8	1110	DB06144	–6.9	–6.7
1064	DB13701	–6.9	–6.8	1111	DB08634	–6.9	–6.7
1065	DB15418	–6.9	–6.8	1112	DB12354	–6.9	–6.7
1066	DB00719	–6.9	–6.7	1113	DB13956	–6.9	–6.7
1067	DB03031	–6.9	–6.7	1114	DB17129	–6.9	–6.7
1068	DB05936	–6.9	–6.7	1115	DB01068	–6.9	–6.7
1069	DB11614	–6.9	–6.7	1116	DB01420	–6.9	–6.7
1070	DB11632	–6.9	–6.7	1117	DB01471	–6.9	–6.7
1071	DB13317	–6.9	–6.7	1118	DB06346	–6.9	–6.7
1072	DB14716	–6.9	–6.7	1119	DB11577	–6.9	–6.7
1073	DB15456	–6.9	–6.7	1120	DB16967	–6.9	–6.7
1074	DB16111	–6.9	–6.7	1121	DB03383	–6.9	–6.7
1075	DB02873	–6.9	–6.7	1122	DB04612	–6.9	–6.7
1076	DB04796	–6.9	–6.7	1123	DB06166	–6.9	–6.7
1077	DB08166	–6.9	–6.7	1124	DB07226	–6.9	–6.7
1078	DB11730	–6.9	–6.7	1125	DB07336	–6.9	–6.7

Table S1. *Continued.*

No.	Compound Code	Docking Score (kcal/mol)		No.	Compound Code	Docking Score (kcal/mol)	
		Quick	Moderate			Quick	Moderate
1126	DB07941	–6.9	–6.7	1173	DB08486	–6.8	–6.6
1127	DB08527	–6.9	–6.7	1174	DB12474	–6.8	–6.6
1128	DB09215	–6.9	–6.7	1175	DB12946	–6.8	–6.6
1129	DB11816	–6.9	–6.7	1176	DB15841	–6.8	–6.6
1130	DB11941	–6.9	–6.7	1177	DB08043	–6.8	–6.6
1131	DB00524	–6.9	–6.7	1178	DB08092	–6.8	–6.6
1132	DB06393	–6.9	–6.7	1179	DB13491	–6.8	–6.6
1133	DB06412	–6.9	–6.7	1180	DB13579	–6.8	–6.6
1134	DB07155	–6.9	–6.7	1181	DB15954	–6.8	–6.6
1135	DB07507	–6.9	–6.7	1182	DB00443	–6.8	–6.6
1136	DB09355	–6.9	–6.7	1183	DB00952	–6.8	–6.6
1137	DB12047	–6.9	–6.7	1184	DB01782	–6.8	–6.6
1138	DB15099	–6.9	–6.7	1185	DB06994	–6.8	–6.6
1139	DB16296	–6.9	–6.7	1186	DB07728	–6.8	–6.6
1140	DB04341	–6.9	–6.7	1187	DB07845	–6.8	–6.6
1141	DB04652	–6.9	–6.7	1188	DB08241	–6.8	–6.6
1142	DB04892	–6.9	–6.7	1189	DB12573	–6.8	–6.6
1143	DB07138	–6.9	–6.7	1190	DB14540	–6.8	–6.6
1144	DB00319	–6.9	–6.7	1191	DB00367	–6.8	–6.6
1145	DB00522	–6.9	–6.7	1192	DB06240	–6.8	–6.6
1146	DB07141	–6.9	–6.7	1193	DB07933	–6.8	–6.6
1147	DB07457	–6.9	–6.7	1194	DB13767	–6.8	–6.6
1148	DB07618	–6.9	–6.7	1195	DB13927	–6.8	–6.6
1149	DB08123	–6.9	–6.7	1196	DB15221	–6.8	–6.6
1150	DB12805	–6.9	–6.7	1197	DB02914	–6.8	–6.6
1151	DB01222	–6.9	–6.6	1198	DB04083	–6.8	–6.6
1152	DB05932	–6.9	–6.6	1199	DB06871	–6.8	–6.6
1153	DB06964	–6.9	–6.6	1200	DB07467	–6.8	–6.6
1154	DB08709	–6.9	–6.6	1201	DB12188	–6.8	–6.6
1155	DB09123	–6.8	–6.6	1202	DB13208	–6.8	–6.6
1156	DB02247	–6.8	–6.6	1203	DB14681	–6.8	–6.6
1157	DB03802	–6.8	–6.6	1204	DB00455	–6.8	–6.6
1158	DB06927	–6.8	–6.6	1205	DB07934	–6.8	–6.6
1159	DB07186	–6.8	–6.6	1206	DB03671	–6.8	–6.6
1160	DB08056	–6.8	–6.6	1207	DB04014	–6.8	–6.6
1161	DB08174	–6.8	–6.6	1208	DB06307	–6.8	–6.6
1162	DB11681	–6.8	–6.6	1209	DB06622	–6.8	–6.6
1163	DB11750	–6.8	–6.6	1210	DB14970	–6.8	–6.6
1164	DB12127	–6.8	–6.6	1211	DB12685	–6.8	–6.6
1165	DB15265	–6.8	–6.6	1212	DB14923	–6.8	–6.6
1166	DB00533	–6.8	–6.6	1213	DB06460	–6.8	–6.5
1167	DB00764	–6.8	–6.6	1214	DB11184	–6.8	–6.5
1168	DB01964	–6.8	–6.6	1215	DB11954	–6.8	–6.5
1169	DB06730	–6.8	–6.6	1216	DB12065	–6.8	–6.5
1170	DB07326	–6.8	–6.6	1217	DB01834	–6.8	–6.5
1171	DB07360	–6.8	–6.6	1218	DB12717	–6.8	–6.5
1172	DB08423	–6.8	–6.6	1219	DB14568	–6.8	–6.5

Table S1. *Continued.*

No.	Compound Code	Docking Score (kcal/mol)		No.	Compound Code	Docking Score (kcal/mol)	
		Quick	Moderate			Quick	Moderate
1220	DB00246	-6.8	–6.5	1261	DB00764	–6.8	−6.0
1221	DB03885	−6.8	–6.5	1262	DB01964	–6.8	−6.0
1222	DB00523	−6.8	–6.5	1263	DB06730	–6.8	−6.0
1223	DB05255	−6.8	–6.5	1264	DB07326	–6.8	−6.0
1224	DB01599	−6.8	–6.5	1265	DB07360	–6.8	−6.0
1225	DB01418	−6.8	–6.5	1266	DB08423	–6.8	−6.0
1226	DB06897	−6.8	–6.5	1267	DB08486	–6.8	−6.0
1227	DB08079	−6.8	–6.5	1268	DB12474	–6.8	−5.9
1228	DB11841	−6.8	–6.5	1269	DB12946	–6.8	−5.9
1229	DB14867	−6.8	–6.5	1270	DB15841	–6.8	−5.9
1230	DB11962	−6.8	–6.4	1271	DB08043	–6.8	−5.9
1231	DB11986	−6.8	–6.4	1272	DB08092	–6.8	−5.9
1232	DB13919	−6.8	–6.4	1273	DB13491	–6.8	−5.9
1233	DB11855	−6.8	–6.4	1274	DB13579	–6.8	−5.9
1234	DB09238	−6.8	–6.4	1275	DB15954	–6.8	−5.9
1235	DB15239	−6.8	–6.4	1276	DB00443	–6.8	−5.8
1236	DB14641	−6.8	–6.4	1277	DB00952	–6.8	−5.8
1237	DB02220	−6.8	–6.4	1278	DB01782	–6.8	−5.8
1238	DB07334	−6.8	–6.3	1279	DB06994	–6.8	−5.8
1239	DB13717	−6.8	–6.3	1280	DB07728	–6.8	−5.8
1240	DB16837	−6.8	–6.3	1281	DB07845	–6.8	−5.7
1241	DB07456	−6.8	–6.3	1282	DB08241	–6.8	−5.7
1242	DB12462	−6.8	–6.3	1283	DB12573	–6.8	−5.7
1243	DB11794	−6.8	–6.3	1284	DB14540	–6.8	−5.6
1244	DB15029	−6.8	–6.3	1285	DB00367	–6.8	−5.6
1245	DB15638	−6.8	–6.3	1286	DB06240	–6.8	−5.5
1246	DB16122	−6.8	–6.3	1287	DB07933	–6.8	−5.5
1247	DB12557	−6.8	–6.3	1288	DB13767	–6.8	−5.4
1248	DB08003	−6.8	–6.3	1289	DB13927	–6.8	−5.3
1249	DB08147	−6.8	–6.3	1290	DB15221	–6.8	−5.3
1250	DB02169	−6.8	–6.2	1291	DB02914	–6.8	−5.2
1251	DB07250	−6.8	–6.2	1292	DB04083	–6.8	−5.1
1252	DB06202	−6.8	–6.2	1293	DB06871	–6.8	−5.0
1253	DB06334	−6.8	–6.2	1294	DB07467	–6.8	−5.0
1254	DB16823	−6.8	–6.2	1295	DB12188	–6.8	−4.9
1255	DB04890	−6.8	–6.2	1296	DB13208	–6.8	−4.8
1256	DB13506	−6.8	–6.2	1297	DB14681	–6.8	−4.8
1257	DB15343	−6.8	–6.1	1298	DB00455	–6.8	−4.3
1258	DB02432	−6.8	–6.1	1299	DB07934	–6.8	−4.0
1259	DB04632	−6.8	–6.1	1300	DB03671	–6.8	−3.2
1260	DB07131	−6.8	–6.1	1301	DB04014	–6.8	−2.9

[a] Data were arranged according to moderate docking scores.

Table S2. Estimated quick, moderate, and expensive docking scores of the promising 194 drug candidates towards VP35 [a].

No.	Compound Code	Docking Score (kcal/mol)		
		Quick	Moderate	Expensive
	**1D9**	**−6.5**	**−6.4**	**−6.4**
1	DB14875	−9.7	−10.3	−10.7
2	DB07800	−9.6	−10.2	−10.6
3	DB15594	−9.3	−9.4	−10.5
4	DB07424	−9.9	−10.4	−10.5
5	DB08858	−10.5	−10.0	−10.5
6	DB06705	−9.8	−9.6	−10.4
7	DB11183	−9.9	−9.9	−10.3
8	DB12051	−8.5	−9.7	−9.8
9	DB12511	−8.1	−8.8	−9.8
10	DB03146	−9.3	−9.8	−9.7
11	DB09267	−8.6	−9.9	−9.6
12	DB04285	−8.4	−9.3	−9.4
13	DB06448	−8.4	−9.4	−9.4
14	DB03104	−8.4	−9.2	−9.3
15	DB00743	−8.7	−8.9	−9.3
16	DB02322	−7.0	−9.0	−9.3
17	DB05340	−8.9	−9.2	−9.2
18	DB02015	−8.2	−8.9	−9.2
19	DB04495	−9.0	−9.2	−9.2
20	DB16637	−8.4	−9.0	−9.2
21	DB17270	−8.3	−8.9	−9.2
22	DB01988	−9.0	−9.2	−9.1
23	DB16262	−8.1	−8.2	−9.1
24	DB01897	−9.0	−9.1	−9.1
25	DB13911	−9.1	−9.1	−9.1
26	DB04049	−8.9	−9.0	−9.0
27	DB11493	–9.3	–9.4	−9.0
28	DB03948	–9.4	–9.4	−9.0
29	DB09335	–9.2	–9.2	−9.0
30	DB15310	–8.8	–8.9	−9.0
31	DB09275	–8.8	–8.9	−9.0
32	DB11611	–8.9	–9.0	−9.0
33	DB15291	–8.8	–8.9	−9.0
34	DB14779	–8.8	–9.0	−8.9
35	DB15385	–8.7	–8.8	−8.9
36	DB02723	–8.7	–8.9	−8.9
37	DB06367	–8.8	–8.9	−8.9
38	DB15193	–8.7	–8.9	−8.9
39	DB03076	–8.5	–8.7	−8.9
40	DB04408	–8.4	–8.6	−8.9
41	DB06595	–8.7	–8.9	−8.9
42	DB02353	–8.5	–8.7	−8.8
43	DB00872	–8.7	–8.8	−8.8
44	DB15416	–8.7	–8.9	−8.8

Table S2. *Continued.*

No.	Compound Code	Docking Score (kcal/mol)		
		Quick	Moderate	Expensive
45	DB00563	–8.6	–8.8	−8.8
46	DB02033	–8.6	–8.7	−8.8
47	DB15068	–8.6	–8.8	−8.8
48	DB09138	–8.6	–8.8	−8.8
49	DB15587	–8.6	–8.8	−8.8
50	DB17109	–8.5	–8.7	−8.8
51	DB04032	–8.4	–8.6	−8.8
52	DB07460	–8.5	–8.6	−8.7
53	DB14950	–8.5	–8.6	−8.7
54	DB15047	–8.4	–8.6	−8.7
55	DB03433	–8.3	–8.4	−8.7
56	DB15822	–8.5	–8.7	−8.7
57	DB02329	–8.5	–8.6	−8.6
58	DB07875	–8.4	–8.5	−8.6
59	DB17059	–8.3	–8.4	−8.6
60	DB07062	–8.4	–8.5	−8.6
61	DB00153	–8.4	–8.5	−8.6
62	DB05075	–8.0	–8.1	−8.6
63	DB17197	–8.3	–8.4	−8.6
64	DB11591	–8.2	–8.3	−8.6
65	DB11977	–8.4	–8.5	−8.6
66	DB14883	–8.4	–8.5	−8.5
67	DB15075	–8.3	–8.4	−8.5
68	DB03358	–8.4	–8.5	−8.5
69	DB06251	–8.1	–8.2	−8.5
70	DB09319	–8.1	–8.1	−8.5
71	DB17021	–8.4	–8.5	−8.5
72	DB06974	–8.3	–8.4	−8.5
73	DB13943	–8.4	–8.5	−8.5
74	DB16117	–7.9	–8.0	−8.5
75	DB16255	–8.3	–8.4	−8.4
76	DB02331	–8.5	–8.6	−8.4
77	DB12228	–8.3	–8.4	−8.4
78	DB15688	–8.0	–8.1	−8.4
79	DB14208	–8.3	–8.3	−8.4
80	DB16844	–8.3	–8.4	−8.4
81	DB00984	–8.3	–8.4	−8.4
82	DB15614	–7.9	–8.1	−8.4
83	DB16244	–8.5	–8.6	−8.4
84	DB07691	–8.3	–8.4	−8.4
85	DB14653	–8.3	–8.3	−8.4
86	DB12424	–8.3	–8.4	−8.4
87	DB14664	–8.3	–8.4	−8.4
88	DB02137	–8.3	–8.4	−8.4
89	DB02388	–7.9	–8.0	−8.4
90	DB05608	–8.3	–8.4	−8.4
91	DB12649	–8.3	–8.4	−8.4

Table S2. *Continued.*

No.	Compound Code	Docking Score (kcal/mol)		
		Quick	Moderate	Expensive
92	DB05410	–8.1	–8.2	−8.4
93	DB12556	–8.3	–8.3	−8.4
94	DB05490	–8.3	–8.3	–8.3
95	DB15821	–8.2	–8.2	–8.3
96	DB16758	–8.3	–8.3	–8.3
97	DB05263	–8.2	–8.3	–8.3
98	DB02809	–9.6	–9.4	–8.3
99	DB04288	–8.2	–8.3	–8.3
100	DB06883	–8.2	–8.3	–8.3
101	DB07872	–8.1	–8.2	–8.3
102	DB11800	–8.2	–8.3	–8.3
103	DB08173	–8.2	–8.3	–8.3
104	DB13640	–8.2	–8.3	–8.3
105	DB15273	–8.1	–8.2	–8.3
106	DB08387	–8.2	–8.3	–8.3
107	DB16047	–8.2	–8.3	–8.3
108	DB03084	–8.2	–8.3	–8.3
109	DB07700	–8.2	–8.3	–8.3
110	DB11809	–8.2	–8.3	–8.3
111	DB12336	–8.3	–8.4	–8.3
112	DB12067	–8.2	–8.2	–8.3
113	DB13947	–8.1	–8.2	–8.3
114	DB01395	–8.2	–8.2	–8.2
115	DB11742	–8.1	–8.2	–8.2
116	DB12877	–8.1	–8.1	–8.2
117	DB12522	–8.2	–8.2	–8.2
118	DB02741	–8.1	–8.2	–8.2
119	DB07833	–8.1	–8.2	–8.2
120	DB08143	–8.0	–8.1	–8.2
121	DB00210	–8.1	–8.2	–8.2
122	DB08962	–8.1	–8.2	–8.2
123	DB13954	–8.1	–8.2	–8.2
124	DB07136	–8.1	–8.2	–8.2
125	DB14209	–8.0	–8.1	–8.2
126	DB14541	–7.9	–8.0	–8.2
127	DB11805	–8.1	–8.2	–8.2
128	DB13014	–8.1	–8.2	–8.2
129	DB06435	–8.1	–8.1	–8.2
130	DB12640	–8.0	–8.1	–8.2
131	DB07145	–8.1	–8.2	–8.2
132	DB15034	–8.0	–8.1	–8.2
133	DB16038	–8.1	–8.1	–8.2
134	DB02852	–8.0	–8.1	–8.1
135	DB04698	–8.1	–8.1	–8.1
136	DB11691	–8.0	–8.1	–8.1
137	DB17117	–7.9	–8.0	–8.1
138	DB07817	–8.0	–8.1	–8.1

Table S2. *Continued.*

No.	Compound Code	Docking Score (kcal/mol)		
		Quick	Moderate	Expensive
139	DB16184	–8.0	–8.1	–8.1
140	DB06997	–8.0	–8.1	–8.1
141	DB11995	–7.9	–8.1	–8.1
142	DB12121	–8.0	–8.1	–8.1
143	DB07252	–8.1	–8.2	–8.1
144	DB14125	–7.9	–8.1	–8.1
145	DB14765	–7.9	–8.1	–8.1
146	DB15039	–8.0	–8.1	–8.1
147	DB16256	–8.1	–8.2	–8.1
148	DB16875	–8.0	–8.1	–8.1
149	DB01134	–8.0	–8.1	–8.1
150	DB14659	–8.1	–8.2	–8.1
151	DB15585	–8.0	–8.1	–8.1
152	DB00351	–8.0	–8.1	–8.1
153	DB01990	–8.1	–8.0	–8.1
154	DB07607	–7.9	–8.1	–8.1
155	DB14045	–7.9	–8.1	–8.1
156	DB15775	–8.0	–8.1	–8.1
157	DB04839	–7.9	–8.1	–8.1
158	DB12024	–8.0	–8.1	–8.1
159	DB12355	–7.9	–8.1	–8.1
160	DB00562	–7.9	–8.1	–8.1
161	DB08386	–7.9	–8.1	–8.1
162	DB14038	–7.9	–8.1	–8.1
163	DB01993	–7.9	–8.0	–8.1
164	DB16272	–7.9	–8.0	–8.1
165	DB00169	–7.9	–8.0	–8.0
166	DB04038	–8.0	–8.1	–8.0
167	DB13664	–7.9	–8.0	–8.0
168	DB14976	–7.9	–8.0	–8.0
169	DB15903	–7.9	–8.0	–8.0
170	DB08232	–7.9	–8.0	–8.0
171	DB12513	–7.9	–8.0	–8.0
172	DB13830	–7.9	–8.0	–8.0
173	DB14632	–8.0	–8.1	–8.0
174	DB02545	–7.9	–8.0	–8.0
175	DB12764	–7.9	–8.0	–8.0
176	DB12756	–7.9	–8.0	–8.0
177	DB16024	–9.2	–9.2	–8.0
178	DB01411	–8.6	–8.8	–7.9
179	DB01761	–8.6	–8.7	–7.9
180	DB12388	–8.0	–8.1	–7.8
181	DB15356	–8.3	–8.3	–7.8
182	DB03932	–8.1	–8.0	–7.7
183	DB03642	–8.2	–8.3	–7.7
184	DB12491	–8.6	–8.3	–7.7
185	DB09233	–8.2	–8.2	–7.5

Table S2. *Continued.*

No.	Compound Code	Docking Score (kcal/mol)		
		Quick	Moderate	Expensive
186	DB04859	–8.4	–8.5	–7.4
187	DB02633	–8.3	–8.4	–7.3
188	DB03231	–8.1	–8.2	–6.8
189	DB08180	–8.8	–9.0	–6.8
190	DB01166	–8.0	–8.1	–6.8
191	DB00471	–7.9	–8.0	–6.4
192	DB02051	–8.1	–8.2	–5.9
193	DB11651	–8.3	–8.3	–5.8
194	DB08353	–7.9	–8.0	–5.3

[a] Data were arranged according to expensive docking scores.

Table S3. Estimated quick, moderate, and expensive docking scores and MM/GBSA binding energies (in kcal/mol) over 25 ns MD simulations of the promising 26 drug candidates towards VP35 [a].

**No.**	**Compound Code**	**Docking Score (kcal/mol)**			**MM/BSA Binding Energy (kcal/mol)**
		**Quick**	**Moderate**	**Expensive**	
	**1D9**	**−6.5**	**−6.4**	**−6.4**	−27.6
1	DB14875	−9.7	−10.3	−10.7	−38.0
2	DB07800	−9.6	−10.2	−10.6	−36.1
3	DB04285	−8.4	−9.3	−9.4	−35.3
4	DB16637	−8.4	−9.0	−9.2	−33.2
5	DB15594	−9.3	−9.4	−10.5	−32.3
6	DB02015	−8.2	−8.9	−9.2	−31.9
7	DB16262	−8.1	−8.2	−9.1	−31.8
8	DB06705	−9.8	−9.6	−10.4	−31.6
9	DB12051	−8.5	−9.7	−9.8	−29.8
10	DB06448	−8.4	−9.4	−9.4	−29.5
11	DB03104	−8.4	−9.2	−9.3	−28.6
12	DB01988	−9.0	−9.2	−9.1	−26.8
13	DB03146	−9.3	−9.8	−9.7	−26.3
14	DB02322	−7.0	−9.0	−9.3	−25.8
15	DB04495	−9.0	−9.2	−9.2	−25.8
16	DB09267	−8.6	−9.9	−9.6	−25.6
17	DB08858	−10.5	−10.0	−10.5	−25.5
18	DB12511	−8.1	−8.8	−9.8	−25.4
19	DB05340	−8.9	−9.2	−9.2	−25.2
20	DB07424	−9.9	−10.4	−10.5	−25.2
21	DB11183	−9.9	−9.9	−10.3	−23.9
22	DB01897	−9.0	−9.1	−9.1	−23.9
23	DB17270	−8.3	−8.9	−9.2	−21.9
24	DB04049	−8.9	−9.0	−9.0	−21.9
25	DB13911	−9.1	−9.1	−9.1	−19.5
26	DB00743	−8.7	−8.9	−9.3	−18.6

[a] Data were arranged according to the MM/GBSA binding energy over 25 ns MD simulations.

Table S4. Estimated quick, moderate, and expensive docking scores and MM/GBSA binding energies (in kcal/mol) over 25 and 50 ns MD simulations of the promising 11 drug candidates towards VP35 [a].

No.	Compound Code	Docking Score (kcal/mol)			MM/GBSA Binding Energy (kcal/mol)	
		Quick	Moderate	Expensive	25 ns	50 ns
	**1D9**	**−6.5**	**−6.4**	**−6.4**	**−27.6**	**−27.9**
1	DB14875	−9.7	−10.3	−10.7	−38.0	−38.3
2	DB07800	−9.6	−10.2	−10.6	−36.1	−36.0
3	DB15594	−9.3	−9.4	−10.5	−32.3	−30.8
4	DB02015	−8.2	−8.9	−9.2	−31.9	−29.8
5	DB16262	−8.1	−8.2	−9.1	−31.8	−29.6
6	DB06705	−9.8	−9.6	−10.4	−31.6	−29.4
7	DB04285	−8.4	−9.3	−9.4	−35.3	−27.5
8	DB16637	−8.4	−9.0	−9.2	−33.2	−27.3
9	DB12051	−8.5	−9.7	−9.8	−29.8	−25.2
10	DB06448	−8.4	−9.4	−9.4	−29.5	−25.1
11	DB03104	−8.4	−9.2	−9.3	−28.6	−25.1

[a] Data were arranged according to the MM/GBSA binding energy over 50 ns MD simulations.

Table S5. The computed MM/GBSA binding energy in triplicate for the most potent drug candidates complexed with VP35 over 250 ns MDS in triplicates.

**No.**	**Compound Code**	**MM/GBSA Binding Energy (kcal/mol)**		
		**1st**	**2nd**	**3rd**
	**1D9**	−29.3	−29.2	−29.2
1	DB14875	−36.6	−36.2	−36.5
2	DB07800	−35.6	−35.5	−35.5
